# Can We Modify the Intrauterine Environment to Halt the Intergenerational Cycle of Obesity?

**DOI:** 10.3390/ijerph9041263

**Published:** 2012-04-16

**Authors:** Kristi B. Adamo, Zachary M. Ferraro, Kendra E. Brett

**Affiliations:** 1 Healthy Active Living and Obesity Research Group, Children’s Hospital of Eastern Ontario Research Institute, Ottawa, ON K1H 8L1, Canada; Email: zach.ferraro@gmail.com (Z.M.F.); kebrett@cheo.on.ca (K.E.B.); 2 Faculty of Medicine, Pediatrics, University of Ottawa, Ottawa, ON K1H 8L1, Canada; 3 Faculty of Health Sciences, School of Human Kinetics, University of Ottawa, Ottawa, ON K1H 8L1, Canada

**Keywords:** child obesity, pregnancy, gestational weight gain, lifestyle change

## Abstract

Child obesity is a global epidemic whose development is rooted in complex and multi-factorial interactions. Once established, obesity is difficult to reverse and epidemiological, animal model, and experimental studies have provided strong evidence implicating the intrauterine environment in downstream obesity. This review focuses on the interplay between maternal obesity, gestational weight gain and lifestyle behaviours, which may act independently or in combination, to perpetuate the intergenerational cycle of obesity. The gestational period, is a crucial time of growth, development and physiological change in mother and child. This provides a window of opportunity for intervention via maternal nutrition and/or physical activity that may induce beneficial physiological alternations in the fetus that are mediated through favourable adaptations to *in utero *environmental stimuli. Evidence in the emerging field of epigenetics suggests that chronic, sub-clinical perturbations during pregnancy may affect fetal phenotype and long-term human data from ongoing randomized controlled trials will further aid in establishing the science behind ones predisposition to positive energy balance.

## 1. Introduction—What is the Problem?

Child obesity is a global epidemic [1]. A dramatic rise in pediatric overweight/obesity (OW/OB) has been evident over the last three decades. In Canada this accounts for 26% of 2–17 year old Canadian children and youth [2]. The battle against child obesity is a high priority throughout the world from both a health care economics and population health perspective. Unfortunately, obesity tracks very closely from childhood to adolescence to adulthood. Over two thirds of obese children will become obese adults [3,4,5]. Moreover, six in 10 obese children have at least one risk factor for cardiovascular disease, and an additional 25% have two or more risk factors [6]. Co-morbidities such as Type 2 diabetes and non-alcoholic fatty liver disease, once considered adult problems, are now reported at a greater frequency among youth [7,8,9,10]. This leads to a greater risk of health complications associated with early morbidity affecting optimal childhood development and quality of life. Consequently, the long-term health care burden is extraordinary. It has been projected that the current generation of children will be the first in modern history to see a shorter life-expectancy than their parents [11] and we know that once it has developed, obesity is very difficult to treat making *early* prevention of paramount importance. We would hazard to say that gestation is the ideal period for preventive efforts, as it is the most critical phase of growth and development experienced throughout the lifespan (*i.e.*, two cells to fully formed human in nine months). Small lifestyle modifications during gestation, that alter the intrauterine environment, may produce substantial changes in health outcomes of the child, thus identifying pregnancy as a critical period to intervene in the development of childhood obesity.

## 2. Why Are We Concerned About Mom?

### 2.1. Maternal Obesity

Epidemiological data from the United States (U.S.) illustrated that between 1993 and 2003, pre-pregnancy obesity increased by 69% (from 13% to 22%) [12] and, over a similar time frame the proportion of women in the obese categories increased from 3% to 10% in the Canadian population [13]. In North America, more than two-thirds of women of childbearing age are overweight (BMI 24.9 to 29.9 kg/m^2^) or obese (BMI > 30 kg/m^2^) [14,15] and the statistics are comparable in the United Kingdom (U.K.) where 53% of women are overweight or obese [16]. This is alarming as children born to overweight or obese mothers are significantly more likely to be large for gestational age (LGA; (birth weight ≥ 90^th^ percentile)) [17,18,19] and obese in infancy and childhood as compared to children of healthy weight mothers [20,21]. In fact, pregravid obesity is the strongest risk factor for childhood obesity and metabolic dysregulation [22].

Birth weight is frequently used as a surrogate marker of the intrauterine environment [23] and a recent meta-analysis by Yu and colleagues, confirmed the association between high birth weight (>4,000 g) and increased risk of downstream obesity (OR 2.07, 95% CI: 1.91–2.24). Subgroup analyses indicated that this relationship persists from preschool age to school age to adolescence and into adulthood [24]. 

**Figure 1:**
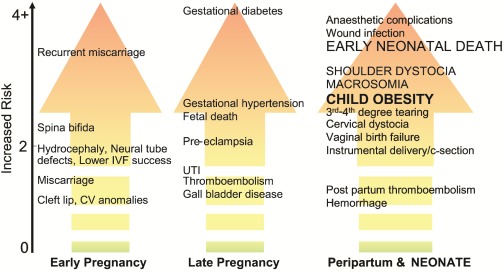
Risks associated with pregnancies complicated by overweight or obesity. The x-axis shows the time course and the y-axis illustrates the degree of elevated risk for each outcome based on published literature (IVF = *in vitro* fertilization, CV = cardiovascular, UTI = urinary tract infection).

This is of particular concern because of the myriad of adverse outcomes (both maternal and fetal) associated with a pregnancy complicated by obesity. To this point, Salihu and colleagues showed that the risk of any form of obstetrical complication was about 3-fold greater in obese *versus* non-obese mothers [25]. While the specific complications are reviewed elsewhere [26,27,28,29,30], Figure 1 illustrates the estimated increased risk for several detrimental sequelae in overweight or obese pregnant women over the course of pregnancy.

Most relevant to this review, maternal overweight/obesity more than doubles the risk of obesity in offspring at 24 months of age [20] and prospective data has shown maternal body mass index (BMI) to be the strongest predictor for both overweight and percentage body fat at 8 yrs of age [22]. Furthermore, rapid growth in the first months of life is associated with increased risk for child overweight [31,32,33,34] and offspring born to overweight mothers are at greater risk for rapid weight gain during first two years of life (OR = 1.22, CI = 0.64–2.32) [35]. Importantly, children with higher range BMIs, as early as 24 months, are more likely to be overweight at age 12 [36]. Maternal pregravid weight status is thus important both clinically, for the health care professional, and from a public health perspective due to the intergenerational nature of obesity.

### 2.2. Gestational Weight Gain

**Figure 2:**
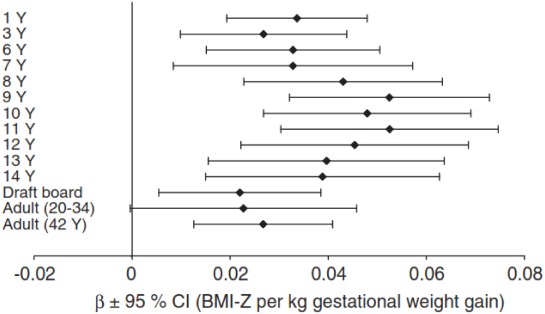
Regression analyses with gestational weight gain as dependent variable and offspring body mass index (BMI) z-scores at different ages as independent variables. Adjusted for sex, maternal age, maternal pre-pregnancy BMI, parental social status at birth, breadwinner’s education, single-mother status, prematurity, edema and smoking during pregnancy. Reprinted from [40] by permission from Macmillan Publishers Ltd.: copyright (2010).

Data from observational studies have shown direct associations between weight gain during pregnancy and birth weight or infant adiposity [37,38]. A confounding factor in most studies has been the inability to separate the genetic and environmental contributions- namely excessive gestational weight gain (GWG) may result in high birth weight because of shared obesity-predisposition genes. In this regard, a recent population-based cohort following over 500,000 women over multiple pregnancies indicated that GWG, in particular excessively high levels of gain, increased birth weight independent of genetic factors [39]. Longitudinal data have also found a strong relationship between GWG and downstream weight status in childhood and through adulthood, regardless of pre-pregnancy weight [40] (Figure 2).

Epidemiological evidence has illustrated the independent effect of GWG on preschool weight and BMI with data indicating that women who gain equal to, or more than the recommended weight during pregnancy, increase their risk of having a child who is overweight by their preschool years [21]. In addition, the odds of offspring overweight at age 7 years have been shown to increase by 3% for every 1 kg of GWG [41]. In the context of GWG, maternal BMI remains a central player as pre-pregnancy BMI delineates the GWG recommendation. The most recent Institute of Medicine (IOM) guidelines recommend a much smaller absolute weight gain range and rate for those categorized as overweight (7–11.5 kg; 0.28 kg/week in the 2nd and 3rd trimester) and obese (5–9 kg; 0.22 kg/week in the 2nd and 3rd trimester) [42] and this is important when discussing ‘excess’ weight gain. Average weight gain in pregnancy has dramatically increased over the last 4 decades from 10 to 15 kg, and data indicate that the mean pregnancy weight gain has increased in all pre-pregnancy BMI categories [43]. While 40% of normal weight women in a U.S.-based women’s health study exceeded the new IOM guidelines, 63% of overweight, and 65% of obese women exceeded the recommendations [44]. Data based on the previous guidelines demonstrated that women who were overweight pre-pregnancy were more likely, by a ratio of nearly 2 to 1, to exceed GWG guidelines than were normal-BMI women [45,46,47,48], however more recent data indicate that overweight women were three times more likely to exceed the recommendations *versus* their normal weight comparators [44]. This is particularly troublesome as population-based studies have suggested the even the stricter GWG guidelines are not sufficiently conservative, and that more restrictive weight gain patterns may optimize maternal and fetal outcomes [49,50,51,52]. For example Hinkle and colleagues have shown that GWG below the IOM guidelines may be beneficial for all obese women, and particularly those women categorized as class II and III. Their recent study indicated that compared with the recommended weight gains of 5–9 kg, a GWG from −4.9 to +4.9 kg decreased the odds of macrosomia and was not associated with SGA [53]. Given that both obesity and GWG are positively associated with infant birth weight [41,49,54,55,56], it is not surprising that the incidence of term babies born LGA has increased dramatically in many countries [57,58,59] over the last few decades.

**Figure 3:**
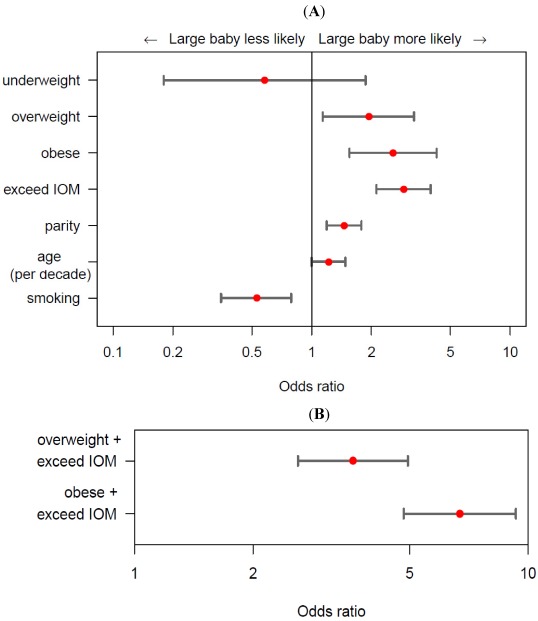
(**A**) Odds ratios and confidence intervals showing the independent contributing factors involved with birthing a LGA age neonate. Analyses were adjusted for gestational age, smoking, parity, and maternal age;(**B**) depicts the joint-association for a women who is either overweight/obese and exceeds IOM recommendations (adapted from [17]).

**Figure 4:**
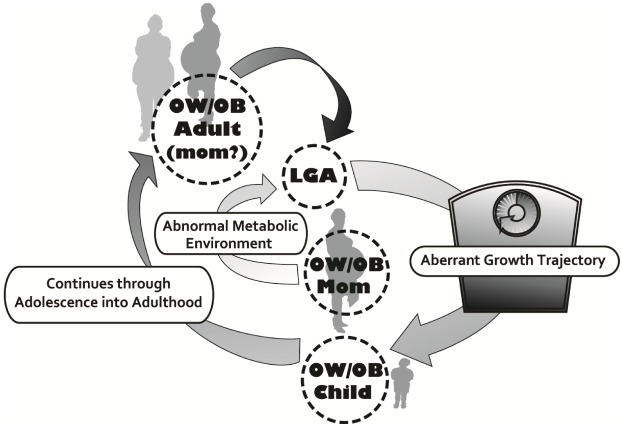
Obesity begets obesity through accelerated growth trajectory without intervention. LGA-large for gestational age, OW/OB-overweight/obese.

For women who are overweight or obese prior to conception, an increase in GWG is correlated with an increase in fetal adiposity [37] and the combination of maternal OW/OB and exceeding GWG guidelines dramatically increases the likelihood of birthing a LGA neonate (Figure 3) [17]. Additionally these women are also very susceptible to post- partum weight retention [60,61,62,63,64], translating to higher rates of post-partum maternal obesity [65], and greater increases in body weight before subsequent pregnancies [62]. This series of events, popularized by Catalano and colleagues [66], is often referred to as the intergenerational cycle of obesity (Figure 4). Interestingly, obesity rates are higher in women worldwide [67] and animal models exploring the intergenerational cycle have shown female offspring of obese dams to be particularly susceptible to downstream obesity [68], thus potentiating this cycle. It is important to clarify that GWG and ensuing postpartum weight retention is not only an issue with women in the higher BMI categories, but an increase in BMI of just 3 kg/m^2^ between two pregnancies increases the risk of GDM, pre-eclampsia, gestational hypertension, C-section delivery, still birth and delivering a LGA neonate even if a woman has a ‘normal’ BMI for both pregnancies [69,70].

Epidemiological studies have shown that mean infant birth weight is highest in women with excessive weight gain during pregnancy, and each 1 kg increment in birth weight increases the odds of overweight in adolescence by 30–50% [18]. It is known that women, regardless of pre-pregnancy BMI status, increase their fat stores in early pregnancy in order to meet the feto-placental and maternal demands of late gestation and lactation [28]. Women who maintain a healthful pre-pregnancy weight generally deposit the majority of this fat centrally in the subcutaneous compartment of the trunk and upper thigh [71,72], however in late pregnancy there is a preferential accumulation of visceral fat [73]. While all women increase their visceral fat stores during pregnancy, there is data to suggest that obese women, who have more subcutaneous fat stores, tend to accumulate more visceral adipose during pregnancy than lean women [71]. The specific fat storage depot is important because of the metabolic differences in fat cell behaviour and visceral adipose tissue is more closely linked to undesirable metabolic outcomes in pregnancy (e.g., GDM, dyslipidemia, hypertension, and pre-eclampsia) [74], and postpartum chronic disease risk status. For example, recent work by our group has indicated that women who retained pregnancy-related weight have a higher level of adiposity many years later (around the menopausal transition) compared to those who return to their pre-pregnancy weight. Furthermore, women who exceeded the GWG guidelines and retained weight after delivery have a higher BMI, % fat and fat mass, fasting insulin level and HOMA score pre-menopause compared to those who gained an excessive amount of weight during pregnancy but returned to their pre-pregnancy weight. These results suggest that returning to pre-pregnancy weight, regardless of GWG, may offer women some protection as they enter menopause [75].

The main criticism for many studies that have identified an association between maternal obesity or pregnancy-related weight gain and downstream child obesity is that these associations might be explained by shared genetic variants or lifestyles between mother and offspring that are related to greater weight gain and adiposity. In response, the association between maternal weight gain (postnatal weight–pre-pregnancy weight) and downstream offspring BMI was examined in a large prospective cohort study of offspring from over 136,000 Swedish families [76]. To tease apart the shared familial and intrauterine contributions, the authors compared within-sibling associations with associations between non-siblings. The authors concluded that, in normal weight mothers, the majority of the association between maternal weight gain and downstream offspring BMI is related to shared familial (genetics and early environmental) characteristics. However, their data suggests a greater contribution of intrauterine mechanisms in overweight or obese women [76]. As a result, weight perturbations during the earliest stages of human development, particularly in the maternally overweight or obese population, can have lifelong impact on adiposity and associated chronic disease.

### 2.3. Gestational Diabetes

While maternal obesity is a well-recognized risk factor for fetal macrosomia, higher pre-pregnancy BMI and higher GWG are also both associated with greater incidence of pre-eclampsia (reviewed elsewhere) [49,77,78] and significantly increased risk of developing gestational diabetes mellitus (GDM) [79,80,81,82], resulting in a metabolically altered fetal environment. For example, maternal hyperglycemia results in fetal hyperglycemia which then leads to excess fetal insulin (*i.e.*, Pedersen hypothesis), and thus fetal overgrowth. However, research has shown that the risk of a woman with well-controlled GDM having a macrosomic baby is related to her pregravid BMI. In comparison to normal-weight women with GDM, overweight women with diet-controlled GDM have been shown to have a 50% greater risk of delivering a macrosomic neonate, while those who are obese have a 2-fold increase. Those who are obese and uncontrolled have a 3-fold greater risk [83,84]. While glucose is thought to be a major contributor to macrosomia and downstream obesity [85,86], studies have shown that lipids (triglycerides and non-esterified fatty acids) are positively correlated with birth weight, [87,88,89] often independent of maternal obesity and glucose, indicating that factors other than glucose are most certainly at play.

### 2.4. Importance of the Intrauterine Environment

Obesity and excessive GWG are thought to change the intrauterine environment and contribute to increased risk of obesity in children. David Barker, whose seminal work initiated a resurgence of the study of fetal programming or developmental plasticity, has stated that ‘*the womb may be more important than the home*’ and encouraged research examining the role of the intrauterine environment on downstream health [90]. Ensuing studies have shown the intrauterine environment to play a critical role in the development of obesity, Type 2 diabetes and the metabolic syndrome in offspring [91,92,93,94,95,96,97]. Historically, the focus of this field was on the relationship between intrauterine growth restriction and downstream health consequences, however several epidemiological studies have highlighted a U or J-shaped relationship between birth weight, adolescent weight, and adult fat mass, finding babies small for gestational age (SGA) and, as mentioned earlier, LGA to be at increased risk [32,98,99,100,101]. Thus the contributions of maternal obesity, and the metabolic impact of fetal overnutrition, elevated birth weight and excess adiposity in neonates has only just begun to garner attention. 

Animal models of human obesity have been particularly useful in further elucidating contributions of obesity and the intrauterine environment on downstream offspring health. In order to identify if maternal obesity is an independent contributor, or if the obesity-inducing behaviour (*i.e.*, obesogenic lifestyle) is responsible for the programming of fetal outcomes, various studies have been performed manipulating only the maternal phenotype or the dietary environment. For example, using a model of overnutrition-driven maternal obesity in Sprague-Dawley rats, Shankar and colleagues examined the metabolic burden on the offspring of exposure to an obese intrauterine environment. The strengths of their model include the use of enteral nutrition for overfeeding thereby bypassing satiety responses, and the ability to exclude parental genetic influences and match for GWG. Additionally, to ensure offspring exposure to obesity was limited to gestation, pups born from the obese dams were cross-fostered to lean dams. Their data illustrate that offspring exposed to maternal obesity *in utero* are more susceptible to obesity, regardless of birth weight, indicating that subtle programming of obesity may occur in the absence of obvious changes in birth weight [68].

Alternatively, to examine the contributions of diet during gestation, Bayol and colleagues examined whether exposure to a maternal junk food diet during pregnancy and lactation influences feeding behaviour in offspring thereby contributing to the development of obesity. Their complex, multi-group experimental design and feeding paradigm demonstrated that, when compared to offspring of dams fed the control diet, rats born to mothers fed the junk food diet during gestation and lactation developed an exacerbated preference for fatty, sugary and salty foods [102]. Interestingly, this research study also showed that a balanced diet during gestation and lactation could provide some protection against junk food diet-induced obesity in offspring. In a follow up study, *in utero* exposure to this same diet was found to exacerbate downstream adiposity and its related metabolic perturbations (glucose, insulin, dyslipidemia) compared to offspring given free access to the junk food diet from weaning but whose mothers were fed a balanced chow diet during pregnancy and lactation. Those exposed to the junk food diet *in utero* but subsequently fed the regular chow diet still exhibited increased fat mass in the major visceral fat pad and adipocyte hypertrophy compared to offspring never exposed, with exposed female offspring presenting a more severe phenotype [103]. These studies illustrate that maternal diet can influence food preferences and feeding responses in offspring and, if not nutritionally sound, can promote adiposity as well as earlier onset of metabolic impairments in offspring.

The closest human approximation illustrating the importance of maternal obesity and the associated intrauterine environment, are those studies examining pregnancies pre- and post- bariatric surgery [104,105]. A study looking at 49 mothers and their 111 offspring demonstrated that, in comparison to their siblings born prior to bariatric surgery, the prevalence of macrosomia was significantly lower in offspring born to women following weight loss surgery (1.8 *vs*. 14.8%) [105]. Additionally, the prevalence of downstream obesity was also notably reduced (3-fold lower) in the offspring of women post-bariatric surgery [105]. Similarly, a study examining 172 children born to 113 women following maternal surgery, found the prevalence of downstream obesity in the offspring decreased by 52% and severe obesity by 45.1%, compared to siblings who were born before maternal surgery [104]. Following surgery, there was also no increase in the prevalence of small for gestational age compared to those born to pre-surgical age and BMI matched women [104].

### 2.5. Epigenetics

The developmental origins of adult disease hypothesis posits that environmental assaults during intrauterine life may alter central regulatory mechanisms of the developing child; an effect thought to be mediated via epigenetic modifications [91,106,107]. Simply put, epigenetics is the environmental influence on gene expression that modifies the genetic message without specifically altering gene sequence. During this critical period, such *in utero *perturbations may alter developmentally plastic systems and predispose the fetus to aberrant movement and ingestive behaviours later in life by compromising physiological thresholds of energy balance regulation [108,109]. As such, chronic exposure to energy surplus, hormones and growth factors *in utero *may potentially increase susceptibility to downstream chronic disease [94,107]. Although considerable animal-model research has illustrated that maternal diet alters offspring body composition associated with epigenetic changes in metabolic control genes [110], there is limited human data investigating the effect of maternal lifestyle on epigenetic modifications. The only human study that has specifically explored the effect of maternal lifestyle on methylation status (*i.e.*, epigenetic changes) illustrated that higher methylation of a specific region of chromosome 9 (RXRA chr9:136355885+) was associated with higher neonatal adiposity and lower maternal carbohydrate intake in early pregnancy. This association between methylation and a mother’s carbohydrate intake raises the possibility that conditions in early pregnancy could affect child’s adiposity through the RXR pathway [111].

Consequently, if greater than 50% of the women of childbearing age are overweight or obese and these pregnant women exceed the weight gain recommendations more often than those of normal weight [61], then maternal BMI may be a key issue related to the short and long term risks for pediatric and adolescent obesity. Taken together, evidence suggests that intervening with the intent to provide a more healthful intrauterine *milieu* is vital to improving health outcomes of mom and baby. Without adequately addressing this critical period we may be compromising the quality of life of the world’s population and placing unnecessary strain on health care systems [112].

## 3. What Can We Do About It?

Knowing that treatment is often unsuccessful once obesity has developed, early prevention efforts are urgently needed. There is no doubt that the seeds of the current obesity crisis facing the adult population were sowed in childhood and as we have purported- likely even earlier. The evidence to date indicates that there are a number of periods in the life course during which there may be specific opportunities to influence behaviour such as critical periods of metabolic plasticity (e.g., early life, pregnancy, menopause), times linked to spontaneous change in behaviour, or periods of significant shifts in attitudes and physiology. Pregnancy is one of these periods when women are motivated to adopt healthy behaviours believing their child may benefit; as evidenced by reduced alcohol consumption and smoking [113,114]. Past efforts to advise women on healthy weight for pregnancy (before, during, and after) have focused less on maternal obesity and more on the concerns about low birth weight delivery outcomes. Although there has been a significant rise in maternal obesity in recent years, *preventing pediatric obesity during pregnancy*, a potential critical period, remains a relatively novel area of study. As such, the acute effects on fetal growth and development *in utero* and subsequent predisposition to obesity in response to maternal dietary intake, physical activity and inactivity, sedentary behaviour, and obesity have not been adequately addressed in the literature.

Over the long-term, children exposed to an intrauterine environment of maternal obesity and born LGA are at increased risk of developing obesity and metabolic syndrome [54,115]. Although weight loss preconception would be ideal in overweight and obese women to prevent this scenario, this recommendation is likely unrealistic given that 49% of pregnancies are unplanned (at least in the U.S.), with 65–75% of these unintended pregnancies being mistimed and 25–35% being unwanted [116]. Knowing that high pre-pregnancy BMI is a primary determinant of GWG [117], and having an obese parent is one of the most significant predictors of childhood obesity [33,34], the World Health Organization [118], Obesity Canada [119], the U.S. Institute of Medicine [120], and the U.K. government [121,122] have all identified childhood obesity prevention as a priority and have acknowledged maternal obesity and the gestational period as primary targets for prevention of downstream childhood obesity. Thus, the gestational period is a crucial time of growth, development and physiological change in mother and child. This provides a window of opportunity for intervention via maternal nutrition and physical activity (PA) that can induce beneficial alternations in fetal physiology mediated through favourable adaptations to environmental stimuli *in utero*. Simply, a healthy, active pregnancy may help to minimize the intergenerational cycle of obesity (Figure 4).

### 3.1. Modifiable Targets—Importance of Physical Activity & Nutrition

**Figure 5:**
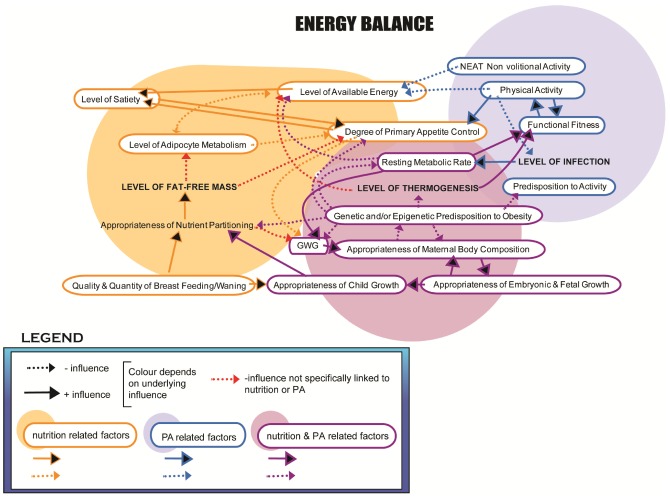
Physiological systems linking maternal obesity and/or adiposity to the development of pediatric obesity.

It is well-established that appropriate nutrition and regular PA are critical mediators of weight gain and weight maintenance at all ages, and they have been specifically identified as predictors of maternal obesity and excessive GWG [45] (Figure 5). In fact, one of the strongest predictors of excessive GWG is higher self-reported caloric intake. Olafsdottir and colleagues showed that those whose GWG exceeded the IOM recommendations consumed, on average, 2,186 calories per day, about 300 calories more than optimal [123]. It is important to clarify that it is not only the caloric intake that is of importance but that the quality of nutrition is equally relevant. The growing fetus obtains all of its nutrients from maternal origins through the placenta, and thus dietary intake has to meet the needs of mom and baby for pregnancy to thrive [124]. While there is an increased requirement of certain vitamins (*i.e.*, A, C, D) and micronutrients (thiamin, riboflavin, folate), the adage of ‘eating for two’ is no longer accepted. We know that changes in metabolism, resulting in more efficient utilization and absorption of nutrients, occur during pregnancy [125,126] and thus the need for increased caloric intake is minimal (~300 kcal in the 3rd trimester) [127]. A balanced maternal diet that is high in fruit and vegetables (*i.e.*, fibre), contains moderate protein from plant and/or animal sources and avoids energy-dense, nutrient poor food choices such as sugar sweetened beverages and saturated fats, not unlike that recommended in the non-pregnant state, is beneficial for both the mother and developing child. Animal model work does suggest that diets high in saturated fat as well as fructose cause insulin resistance in offspring [128,129,130], leading some practitioners to limit fructose consumption in their high risk patients. A modest negative association between maternal BMI and diet quality has been identified [131], and obesity is associated with lower levels of vitamin D [132] and folic acid levels during the childbearing years [133], which may increase the risk of insulin resistance and neural tube defects, respectively [134]. In fact, the findings of Deierlein *et al*. [135] suggest that dietary energy density is a modifiable factor that may assist pregnant women in managing their weight. In their study, compared to women in the reference group consuming foods with a mean energy density of 0.71 kcal per gram, those in the highest quartile (*i.e.*, 1.21 kcal per gram) gained more weight during pregnancy. Furthermore, in comparison to controls, women receiving individualized diet plans catering to their pre-gravid weight, activity level and GWG, had fewer perinatal complications and had infants with lower birth weights, and a lower percentage of LGA and less macrosomia [136], demonstrating that proper nutrient intake during pregnancy has the potential to significantly affect the health of both mother and child.

The available evidence indicates that regular prenatal PA does not increase adverse pregnancy or neonatal outcomes [137,138,139,140,141], but rather is an important component of a healthy pregnancy (see the review by Ferraro, Gaudet and Adamo [142]). Failure to exercise may be associated with decreases in fitness, excessive GWG, varicose veins, lower-back pain, GDM and pregnancy-related high blood pressure [140]. The rise in maternal obesity, in part due to physical inactivity, has been accompanied by an increased prevalence of GDM in women [143,144]. PA has many beneficial physiological effects and regular moderate intensity PA during pregnancy is associated with reduced incidence of GDM [145,146,147,148]. Regular PA with an appropriate rate of progression and intensity, that considers stage of pregnancy and health status of the pregnant woman, has also consistently been shown to reduce the risk of pre-eclampsia [149,150,151,152,153]. Recent consensus in the literature states that regular PA during pregnancy in well-nourished populations is safe and does not negatively impact maternal or neonatal outcomes [154]; however, monitoring fetal growth, maternal weight, nutritional intake and exercise duration and intensity is necessary [140,155]. There is evidence that women who exercise before and during pregnancy tend to weigh less, gain less weight, have improved labour pain tolerance and deliver smaller babies than those who do not [156]. It is not unreasonable to expect that a program combining healthy eating and activity habits would lead to healthy fetal growth and development, resulting in fewer pregnancy-related complications, normal weight offspring, and less maternal weight gain and retention [157].

In fact, PA during pregnancy appears to protect against birth weight extremes (*i.e.*, SGA and LGA) and increases the likelihood of delivering an appropriate for gestational age infant. Most studies have not shown a significant detrimental effect on birth weight with moderate amounts of exercise [158,159,160,161,162,163,164,165,166], suggesting that regular PA is safe and does not compromise fetal growth. Furthermore, a recently published randomized controlled trial concluded that exercise training may attenuate adverse consequences of a pregnancy complicated by overweight or obesity on infant size at birth [167]. The optimization of infant birth weight in women who engage in regular PA is thought to result from an increased functional capacity of the placenta to appropriately deliver nutrients via an increase in placental surface area, improvements in blood flow and an enhanced perfusion balance [168,169]. These findings may be an advantage for overweight or obese pregnant women as a way to reduce their risk of delivering a LGA infant. Observational data from a large birth cohort demonstrated that routine engagement in exercise during pregnancy protects the developing infant from birth weight extremes (*i.e.*, SGA and LGA) [170]. For instance, Clapp noted an asymmetric reduction in birth weight of exercising mothers, a difference that was entirely accounted for by a reduction of neonatal fat mass with no changes in lean mass compared to the offspring of matched controls [171]. However, some studies have demonstrated a link between maternal PA and low birth weight [172,173,174,175]. In these studies, an important limitation was lack of controlling for dietary intake. However, many of these classical studies have focused on lean, healthy active women [168,171,176,177] and as such the reported effects may not be representative of all populations. General physical activity recommendations for a healthy pregnancy have been published [140,178], including those for overweight/obese women [179], and an educational review of the potential benefits of an active pregnancy and simple exercise prescriptions can be found in the literature [142,179].

While results from interventions designed to address modifiable risk factors including unhealthy dietary intake and physical inactivity to improve maternal-fetal outcomes are now being reported, currently, there is limited evidence from well-designed, appropriately powered randomized controlled trials to address the effects on fetal growth outcomes [141]. The inconsistency of results from studies examining the effect of PA on maternal and fetal outcomes likely arises from differences in the type, frequency, timing and duration of the activity program imposed [180]. Further, lack of control for confounding variables including maternal nutritional status (e.g., gestational caloric intake), gestational age at birth and socio-economic status may also contribute to the discrepancy in the literature [141]. Overall, it is important to understand the complex interaction between maternal obesity, GWG, dietary intake and PA to properly address both sides of the energy balance equation when designing and implementing efficacious intervention strategies for maternal-fetal benefit. 

### 3.2. Pregnancy Specific Interventions

As reviewed in Table 1, there have been approximately 35 intervention studies published, of varying design, sample size and success, that have intervened during the gestational period.

Other than the most recently published interventions, most of these individual studies have been considered in the nine relevant systematic or meta-analytic reviews [181,182,183,184,185,186,187,188,189] and one comprehensive review [190] addressing the issue of weight-management interventions during pregnancy or the postpartum period. Interestingly each review, even those completed over the same time frame, included a slightly different combination of papers as identified in Table 1, likely due to the subtle differences in the objectives or aims of each review. The overall findings of these specific reviews are discussed in the following section.

**Table 1:** Behaviour intervention trials targeting the gestational period.

Author	Population	Objective	Intervention	Primary Outcome	Findings: Maternal Outcome	Findings: Neonatal Outcome
**27 RCTs**						
Rae 2000 [191] **f**	GDM population Australia 110% ideal BMI n (I) = 66 n (C) = 58	To identify if treatment of obese women with GDM could reduce insulin therapy and incidence of macrosomia	**Nutrition** Energy restriction diet (70% of recommended intake)	Need for maternal insulin therapy & infant macrosomia	No difference in requirement for insulin (but trend toward need later in pregnancy and for lower dose in intervention)	No difference in BW
Clapp 2000 [176] **h**	Sedentary, non-overweight n (I) = 22 n (C) = 24	To identity the effect of beginning moderate-intensity exercise in early pregnancy on fetoplacental growth	**Exercise** 20 min of monitored, weight-bearing activity 3–5 times/wk @ 55–60% of VO2max (treadmill, step aerobics or stair stepper)	Antenatal placental growth Neonatal and placental morphometry	No difference in GWG	No difference in gestational age. BW & length > in exercise group because of > lean body mass, lower % BF Placenta: exercisers > growth rate & volume, and > functional volume
Marquez-Sterling 2000 [192] **h**	Sedentary non-obese primigravida USA n (I) = 9 n (C) = 6	To examine the effects of exercise on physical and psychological variables	**Exercise** 3-1 h supervised sessions/wk ‘aggressive’ aerobic training; combination of rowing, cycling, walking/jogging, rhythmic calisthetics and step classes		Significant improvement in aerobic fitness (*p* = 0.035) Improvement in several scores on the Body Cathexis Scale (*p* < 0.05) No difference in GWG or body composition	No difference in BW or APGAR
Polley 2002 [193] **a,b,c,d,e,f,i**	Low-income USA BMI > 19.8 Age > 18 years n (I) = 57 n (C) = 53	To determine whether a stepped care, behavioral intervention will decrease the percentage of women who exceed the 1990 IOM GWG recommendation.	**Nutrition & Exercise ** Stepped-care behavioural counseling sessions at prenatal appointments re: recommended GWG, nutrition & exercise. Provision of personalised graph of weight gain trajectory.Bi-weekly education re: healthy eating and exercise delivered via mail	Reduce proportion of women who exceed GWG recommendations	Overall no significant difference. *Normal weight subgroup*: significant reduction in GWG reduction in those exceeding 1990 IOM recommendations (*p* < 0.05). *Overweight subgroup*: Opposite trend overweight women (32 *versus* 59%, *p* = 0.09).	No difference in BW or complications during pregnancy/delivery
Bechtel-Blackwell 2002 [194] **b **	African-American teens USA Age 13–18 years n (I) = 22 n (C) = 24	To conduct computer-assisted self-interview (CASI) nutrition assessment in pregnant, adolescents to compare the effect of a nutrition education intervention with the standard dietitian consult on GWG patterns and postpartum weight retention.	**Nutrition** Patient education. Group sessions. Repeated nutritional assessment.	Reduction in GWG and PPWR at 6 weeks	1st trimester; less GWG (*p* < 0.000) 2nd trimester; no difference (*p* = 0.056) 3rd trimester; higher GWG (*p* < 0.006) higher PPWR in control group at 6 weeks (*p* < 0.0024)	
Prevedel 2003 [195] **h** Prospective, random cohort study	low-risk nulliparous Brazil n (I)=22 n (C ) =19	Aimed to study maternal (body composition andcardiovascular capacity) and perinatal (weight and prematurity) effects of hydrotherapy during pregnancy	**Exercise** Hydrotherapy throughout gestation	Maternal body composition and cardiovascular capacity. Perinatal weight and Prematurity.	Intervention group maintained their fat index and VO2 max. Control group increase their fat and saw a reduction in VO2max.	No difference in prematurity or weight loss in newborns
Barakat 2008 [196]	Sedentary gravidae Caucasian Spain n (I) = 72 n (C ) = 70	This study aimed to determine the possible cause–effect relationship between regular exercise during the 2nd and3rd trimesters of pregnancy by previously sedentary, healthy gravidae and gestational age at the moment of delivery	**Exercise ** The supervised training programme focused mainly on very light resistance and toning exercises and included ,80 sessions (three times/week, 35 min/session from weeks 12–13 to weeks 38–39 of pregnancy)	Risk of preterm delivery and neonatal APGAR scores		no difference in gestational age or APGAR scores
Barakat 2009 [167,197] **h**	Sedentary gravidae Spain n (I) =80 n (C ) = 80	Examined the effect of light-intensity resistance exercise training performed during the 2nd and3rd trimester of pregnancy by previously sedentary and healthy women on the type of delivery and on the dilation, expulsion, and childbirth time [197] and birth size [167]	**Exercise ** The training programme focused on light resistance and toning exercises (3 times/wk, 35–40 min per session)	Main outcomes were maternal and newborn characteristics, the type of delivery (normal, instrumental, or cesarean), and dilation, expulsion, childbirth time and neonatal size at birth	No difference between groups with regard to delivery type (normal, instrumental, or cesarean) The mean dilation, expulsion, and childbirth time did not differ between groups	No differences between control and intervention in Apgar score, BW, birth length, and head circumference of the newborn
Santos 2005 [198] **h**	OW-BMI 25-30 Brazil n (I) = 37 n (C) = 35	To evaluate the effect of aerobic training on submaximal cardiorespiratory capacity in overweight pregnant women	**Exercise** 3- 1 h aerobic exercise session/wk @ 50-60% max predicted HR never exceeding 140 bpm	Cardiorespiratory fitness	Improvement in VO2 at aerobic threshold (*p* <0.002) Improvement in ventilation at aerobic threshold (*p* = 0.02) No difference in weight or GWG	No difference in BW, prevalence of low BW, premature birth, APGAR
Garshasbi 2005 [199] **h**	Primigravida Mean BMI ~ 26 Iran n (I) = 107 n (C) = 105	To investigate the effect of exercise on the intensity of low back pain and kinematics of the spine	**Exercise** 1 h supervised program 3 ×’s /wk @ < 140 bpm included walking, anaerobic exercise, and other specific strengthening exercises	Prevention or reduction of low back pain	Sign difference in intensity of low back pain favouring exercise Sign reduction in flexibility of spine in both groups but greater reduction in exercising group No difference in GWG or length of pregnancy	No difference in BW
Hui 2006 [200] **a,b,c,d,i**	Socioeconomically deprived women in urban core Canada n (I) = 24 n (C) = 21	To deter mine the feasibility of implementing a community based exercise/dietary intervention program aiming to reduce risks of obesity and diabetes	**Nutrition & Exercise** Group exercise sessions and home-based exercise (3–5 ×/week for 30–45 min per session) also recommended. Video exercise instruction was provided to assist.Intervention also included computer-assisted Food Choice Map dietary interviews and counselling	Improve pregnancy outcomes	No significant difference in GWG or adherence to guidelines PA level sign higher (*p* = 0.005)	No difference in BW
Wolff 2008 [201] **a,b,d,g,i**	Caucasian, non smoking Denmark. BMI > 30 n (I) = 23 n (C) = 27	To investigate whether restriction of GWG in obese women can be achieved via diet counseling	**Nutrition** Individual dietary consultations on 10 separate occasions during pregnancy. Healthful diet instruction and restriction of energy intake	Reduction in pregnancy induced increases in insulin, leptin and glucose	Less GWG in the intervention group (*p* = 0.002) lower energy intake (*p* = 0.001) less perturbation in insulin & leptin (*p* = 0.004) less PPWR in intervention (*p* = 0.003)	
Asbee 2009 [202] **a,b,c,d,i**	USA BMI < 40.5 Age 18–49 years n (I) = 57 n (C) = 43	To estimate whether an organized, consistent program of dietary and lifestyle counseling prevents excessive GWG	**Nutrition & Exercise** 1× consultation with dietician in early pregnancy. (40% CHO, 30% PRO, 30% FAT) Information about IOM recommendations and weight grid provided. Moderate exercise recommended 3–5 ×/wk if not following guidelines—Diet & exercise regime reviewed and modified	Reduce proportion of women who exceed GWG recommendations	Intervention sign < GWG (*p* = 0.01) But no significant difference in adherence to IOM GWG recommendations (*p* = 0.21). No difference in preeclampsia, GDM	Trend for lower c-section rate in intervention (*p* = 0.09) Higher c-section rate in control due to ‘failure to progress’
Jeffries 2009 [203] **a**	Australia n (I) = 125 n (C) = 111	To asses effect of a personalized GWG recommendation with regular measurement on GWG	Women were given optimal GWG range and asked to self-monitor weight at various time points over course of pregnancy	Reduce excessive GWG	Reduced GWG in OW women (*p* = 0.01) No difference in adherence to 1990 GWG guidelines	No difference in gestational age, BW, complications or APGAR score
Thornton 2009 [204] **g,i**	OB-BMI > 30 USA n (I) = 116 n (C)= 116	To assess effect o nutritional intervention (energy restriction) on perinatal outcomes.	**Nutrition** Balanced dietary program with energy restriction and food diary monitoring (18 to 24 kcal/kg balanced nutritional regimen, consisting of 40% CHO, 30% PRO, and 30% FAT; not < 2000 kcal/day)	To reduce negative perinatal outcomes	Reduced GWG (*p* < 0.001) Reduced gestational hypertension, *p* < 0.046 less 6-week PPWR *p* < 0.001 no difference in preeclampsia or GDM	No difference in BW, macrosomia, c-section, APGAR score
Landon 2009 [205]	Mild GDM USA n (I) = 485 n (C) = 473	to determine whether treatment of women with mild GDM reduces perinatal and obstetrical complications	**Nutrition** Formal nutrition counseling and diet therapy, as per the American Diabetes Association’s recommendations and interventions for diabetes. Self-monitoring of blood glucose, and insulin therapy (if necessary)	composite of stillbirth or perinatal death and neonatal complications, including hyperbilirubinemia, hypoglycemia, hyperinsulinemia, and birth trauma	Fewer cesarean deliveries in the treatment group. Lower frequency of pre-eclampsia and gestational hypertension in the treatment group. BMI at delivery and GWG was lower in the treatment group	No significant difference between the groups in the frequency of composite primary perinatal outcome. Mean BW, neonatal fat mass and frequency of LGA and macrosomia was significantly reduced in the treatment group
Baciuk 2008 Cavalcante 2009 [206,207] **h**	Low-risk sedentary Brazil n (I) = 34 n (C) = 37	To evaluate the effectiveness and safety of a water aerobics program for low risk, sedentary pregnant women on the maternal cardiovascular capacity during pregnancy, labor and neonatal outcomes evolution of pregnancy	**Exercise** regular, moderate practice of water aerobics for 50 min, 3 ×/wk @ 70% of predicted max HR	Maternal BMI, GWG, blood pressure, cardiovascular capacity, labour type and duration, mode of delivery and neonatal outcomes (BW, viability)	No difference in GWG, maternal BMI, or % body fat, blood pressure, heart rate, maternal cardiovascular capacity, duration of labour, or the type of delivery between the two groups	No differences in incidence of preterm birth, vaginal births, low BW, or adequate weight for gestation
Ong 2009 [148] **h**	Sedentary, OB women Australia n (I) = 6 n (C ) =6	To investigate the effect of a supervised 10-week, home-based, exercise programme, beginning at week 18 of gestation, on glucose tolerance and aerobic fitness	**Exercise** Intervention—10 weeks of supervised home-based exercise - 3 sessions/wk of stationary cycling.10 min warm-up followed by one or two 15 min bouts of cycling (with rest periods if necessary) at an intensity of 50–60% HRmax. As the weeks progressed, the exercise intensity was increased to 60–70% HRmax, while the duration was increased to 40–45 min	Glucose and insulin responses to an oral glucose tolerance test (OGTT), as well as their aerobic fitness	Exercise had favourable effects on glucose tolerance and fitness in obese pregnant women compared to control	
Guelinckx 2010 [208] **a,c,d,g,i** 3-arm RCT (passive vs. Active vs. Control)	BMI > 29 Non-diabetic Belgium n (I passive) = 65 n (I active) = 65 n (C) = 65	To study whether a lifestyle intervention based on a brochure or on active education can improve dietary habits, increase PA, and reduce GWG in obese pregnant women	**Nutrition & Exercise** Information and counseling re: PA during pregnancy. Group nutritional counseling about healthful eating and nutritionally sound substitutions	Reduction in GWG	No significant difference in GWG or adherence to guidelines	No difference in BW, LGA, c-section rate or infant length
Hopkins 2010 [209] **h**	Nulliparous aged 20–40 yrs New Zealand n (I ) = 47 n (C) = 37	To determine the effects of aerobic exercise training in the second half of pregnancy on maternal insulin sensitivity and neonatal outcomes	**Exercise** home-based stationary cycling 5 ×/week, 40 minutes/session from 20 wk gestation to delivery	Maternal insulin sensitivity, neonatal auxology, body composition, and growth-related peptides in cord blood	No difference in maternal insulin sensitivity	lower birth weight (*p* < 0.03) and body mass index at birth (*p* < 0.04). Exercise offspring had lower cord serum IGF-I (*p* < 0.03) and IGF-II (*p* < 0.04)
Korpi-Hyovalti 2011 [210]	At risk of GDM Finland n (I) = 27 n (C) = 27	To evaluate if a lifestyle intervention during pregnancy is feasible in improving the glucose tolerance of women at a high-risk for GDM	**Nutrition & Exercise** Diet: 50–55% carbohydrate, 15 g fibre/1000kcal, fat 30%, protein 15–20%. 30 kcal/kg/day for normal weight, and 25 kcal/kg/day for OW. Exercise: moderate intensity PA was encouraged during pregnancy and 6 appointments with a physiotherapist to encourage PA.	Maternal glucose tolerance, the incidence of GDM and perinatal complications.	No differences in change in glucose tolerance from baseline to weeks 26–28 of gestation. Trend towards less GWG in the intervention.	Mean BW was higher in the intervention group, but not difference in macrosomia. No differences in neonatal outcomes.
Hui 2011 [211]	Non-diabetic, urban-living Canada <26 wks N (I) = 102 N (C) = 88	To examine the effect of an exercise and dietary intervention during pregnancy on excessive GWG, dietary habits and PA habits	**Nutrition & Exercise** Provided with community-based group exercise sessions, instructed home exercise (total of 3–5 ×/wk) and 2 dietary counseling sessions (upon enrolment and 2 months in)	Reduce prevalence of excessive GWG, levels of PA and dietary intake	After 2 months the intervention group reported lower daily intake of calories, fat, sat. fat, chol. (*p* < 0.01) and higher PA compared with control (*p* < 0.01) Lifestyle intervention reduced excessive GWG (*p* < 0.01)	
Luoto 2011 [212] Cluster RCT	BMI ≥ 25, or GDM or previous macrosomic newborn Finland n (I) = 219 n (C) = 180	To examine if GDM or high BW can be prevented by lifestyle counseling in high risk women.	**Nutrition & Exercise** Individualized counseling on PA (to meet recommendations of 800 MET minutes/wk), healthful diet (high fibre, low fat, low sugar choices) , and GWG at 5 antenatal visits	Incidence of GDM and LGA neonate	No difference in incidence of GDM (ES 1.36, 95% CI: 0.71–2.62, *p* = 0.36)	Lower BW (*p* = 0.008) and proportion of LGA neonates (*p* = 0.042)
Phelan 2011 [213] **i**	Normal weight or OW/OB USA n (I) = 182 n (C) = 176	To examine if a behavioural intervention could reduce the number of women exceeding 1990 GWG guidelines and increase the number of women returning to pregravid weight by 6 months post-partum	**Nutrition & Exercise** One face-to-face visit, weekly mailed educations material promoting appropriate GWG, healthy eating and exercise. After each clinic visit individual GWG graphs were provided and 3, 10-15 min telephone calls from dietitian. Additional calls were placed to those not on track with GWG guidelines	Reduce prevalence of excessive GWG and PPWR	Reduced number of normal weight women exceeded GWG guidelines (*p* = 0.003) Increased number of normal and overweight/obese women who return to the pregravid weight (*p* = 0.005)	
Quinlivan 2011 [214] **g**	BMI ≥ 25 Australia n (I) = 63 n (C) = 61	To evaluate whether a 4-step multidisciplinary protocol of antenatal care for OW and OB women would reduce the incidence of GDM	1. Continuity of care by a single maternity care provider; 2. assessing weight gain at each antenatal visit; 3. brief intervention (5 min) by a food tech before each visit; 4. assess by clinical psych, if difficulties identified, an individualized solution-focused treatment plan was implemented.	Reduce prevalence of combined diagnoses of decreased gestational glucose tolerance and GDM.	Intervention was associated with a sign reduction in incidence of GDM (OR 0.17 95% CI 0.03–0.95, *p* = 0.04).Intervention also assoc with reduced GWG (*p* < 0.0001)	No difference in BW (*p* = 0.16)
Nascimento 2011 [215]	OW/OB-BMI ≥ 26 Gest age: 14-24 wks Brazil n (I) = 40 n (C) = 42	To evaluate the effectiveness and safety of physical exercise in terms of maternal/ perinatal outcomes and the perception of quality of life (QoL)	**Exercise** Weekly exercise class under supervision and received home exercise counseling to be performed 5 ×/wk	Reduction of GWG and proportion exceeding the GWG guidelines.	No difference in absolute GWG or numbers exceeding guidelines (47 *vs*. 57%). No difference in QoL The overweight women in the intervention gained sign. less weight (*p* = 0.001)	
Haakstad 2011 [216,217]	Sedentary, nulliparous Norway n (I) = 52 n (C) = 53	To examine the effect of a supervised exercise-program on birth weight, gestational age at delivery and Apgar-score	**Exercise** - supervised aerobic dance and strength training : 60 minutes, 2 ×/wk for a minimum of 12 wks, + 30 min of self-imposed PA on the non-supervised days. All aerobic activities were performed at moderate intensity or RPE of 12–14 (somewhat hard) on Borg’s scale	BW, gestational age at delivery and APGAR-score	More women in the intervention met GWG guidelines Intervention participants who attended 24 exercise sessions (n = 14) differed significantly from controls with regard to weight gain during pregnancy (*p* < 0.01) and postpartum weight retention (*p* < 0.01)	Intervention was not associated with reduction in BW, preterm birth rate or neonatal well-being
Vinter 2011 [218]	Obese, BMI 30–45 Denmark n (I) = 150 n (C) = 154	To study the effects of lifestyle intervention on gestational weight gain (GWG) and obstetric outcomes.	**Nutrition & Exercise** Individualized dietary counseling at 4 time points to assist in limiting GWG to 5 kg. Encouraged to engage in moderate PA 30–60 min daily. Were provided with a pedometer and a fitness membership for 6 months, which included private classes with an exercise specialist. Women also had 4–6 group meetings with specialist who assisted them with integrating of PA in pregnancy and daily life.	Obstetric and neonatal outcomes: GWG, preeclampsia, pregnancy-induced hypertension (PIH), GDM, cesarean section, macrosomia/large for gestational age (LGA), and admission to neonatal intensive unit.	Significantly lower GWG, *p* = 0.01 Trend for fewer intervention women to exceed IOM recommendations (35% *vs*. 47%, *p* = 0.058) No difference in c-section, pre-eclampsia/PIH, GDM	Higher BW in intervention group (3,742 *vs*. 3,593 g, *p* = 0.039)
**8 Non-RCTs**						
Gray-Donald 2000 [219] **a,b,c** Historical control	Cree First Nations population. Canada. n (I) = 112 n (C) = 107	To evaluate an intervention aimed at improving dietary intake during pregnancy, optimizing GWG, glycemic levels and BW, and avoiding unnecessary PPWR	**Nutrition & Exercise** Exercise/walking groups. Nutrition information re: improving healthful food intake via radio broadcasts, booklets, supermarket tours and cooking demos	Improve dietary I/T, optimize GWG, glycemia, birthweight & PPWR	No sign difference in GWG, glycemic levels, or PPWR	No difference in BW
Olson 2004 [220] **a,b,c** Prospective cohort & Historical control	BMI 19.8–29.0 USA. Age > 18 years n (I) = 179 n (C) = 381	To evaluate the eﬃcacy of an intervention directed at preventing excessive GWG.	**Nutrition & Exercise** Education of healthcare providers. Personalized GWG grid. Participant education about PA by-mail. Dietary ‘health checkbook’ and self-monitoring tips and newsletters	Prevention of excessive GWG	No overall significant difference in GWG (*p* = 0.3). Significant difference in GWG and adherence to guidelines in ‘low-income’ subgroup (*p* = 0.01). Less PPWR in low income OW subgroup	No difference in infant BW
Kinnunen 2007 [221] **a,b,c,i** Controlled trial	Primipara Finland Age > 18 years n (I) = 49 n (C) = 56	To investigate whether individual counselling on diet and physical activity during pregnancy can have positive effects on diet and leisure time physical activity and prevent excessive GWG	**Nutrition & Exercise** Information provided about GWG guidelines. Individual counseling concerning diet (4 sessions) and physical activity (5 sessions). Option to attend group classes	Improve diet and PA and prevention of GWG	No significant difference in total GWG (*p* = 0.77). No significant difference in proportion exceeding IOM recommendations (*p* = 0.053)	Significant difference in BW: 15% LGA in control *vs*. none in intervention (*p* = 0.006)
Claesson 2008 [222] **a,b,c** Prospective case-Historical control	OB-BMI > 30 Sweden n (I) = 155 n (C) = 193	To minimize obese women’s GWG to less than 7 kg and to investigate the delivery and neonatal outcome	**Nutrition & Exercise** CBT Patient education and motivational interview. Frequent individual sessions. Weekly aqua aerobic exercise and information about nutrition during pregnancy	Reduce GWG to <7 kg	Significantly less weight gain in the intervention group (*p* < 0.001) Better adherence to GWG guidelines (*p* = 0.003). No difference in pregnancy outcomes	No difference in mode of delivery
Shirazian 2010 [223] **c** cohort-matched historical control	OB-BMI > 30 USA n (I) = 21 n (C) = 20	To investigate if a comprehensive lifestyle modification program would limit GWG and reduce obesity-related complications	**Nutrition & Exercise** Written material, seminars, and counseling sessions for both encouraging walking (self monitor via pedometer), and healthful eating (food diary, calorie counting)	Reduce GWG	Significantly less GWG in intervention group (*p* = 0.003)	No difference in BW, gestational age at delivery, preeclampsia, gestational HTN, GDM, c-section, fetal complications and labour complications
Mottola 2010 [224] Single arm-historical matched control	OW/OB- BMI ≥ 25 Canada n (I) = 65 n (C) = 260	To determine the effect of a nutrition and exercise program on GWG, birthweight, and PPWR.	**Nutrition & Exercise** Individualized nutrition plan with E/I~2000 kcal/d and walking program 3–4 ×/wk	Prevent excessive GWG, BW and PPWR	80% of intervention women meet GWG recommendations 53% of NELIP women were within 2 kg of pre-pregnancy weight at 2 months post partum	No difference in BW
Lindholm 2010 [225] Prospective intervention No control group	OB-BMI > 30 n = 27	To control GWG among obese women by a dietary and physical activity program	**Nutrition & Exercise** - meeting with midwife bi-weekly - 2 support group sessions - 1 dietary consultation - food diaries & PA diaries - aqua fitness class 1×/wk and encouraged to exercise for 30 min on the other days	To limit GWG to ≤6 kg	- 56% met the goal of ≤6 kg	All AGA babies
Artal 2007 [145] Prospective intervention (self-enrolled)	OB with GDM USA n (Ex+Diet) = 39 n (Diet) n= 57	To examine whether weight gain restriction, with or without exercise, would impact glycemic control, pregnancy outcome and total GWG	**Nutrition & Exercise** All patients were provided a eucaloric or hypocaloric consistent carbohydrate meal plan and instructed in self-monitoring blood glucose. Exercise and diet group prescribed an exercise routine equal to 60% symptom-limited VO2max (1 time/wk supervised in the lab and 6 days/wk independently)	Improved glycemic control, pregnancy outcome and total GWG	Weight gain was significantly lower in subjects in the exercise and diet group No difference in complications or c-section delivery	No difference in gestation age. Fewer macrosomic neonates in moms who restricted intake and exercised

Legend: OW = overweight, OB= obese, RCT = randomized controlled trial, BW= birth weight, GWG = gestational weight gain, I = intervention, C = control, PPWR = post partum weight retention, GDM = gestational diabetes mellitus, QoL = quality of life, PA = physical activity. Considered in published systematic reviews: a= Skouteris *et al*. 2010 [186], b = Ronnberg *et al*. 2010 [185], c =Streuling et al 2010 [187], d = Campbell *et al*. 2011 [181], e = Kuhlmann *et al*. 2008 [183], f = Dodd *et al.* 2008 [182], g = Quilivan *et al*. 2011 [184], h = Streuling *et al*. 2011 [188], i = Tanentsapt *et al*. 2011 [189].

### 3.3. Systematic Reviews

The objective of the systematic review performed by Dodd and colleagues in 2008 [182] was to assess the benefits and harm of dietary and lifestyle interventions during pregnancy to improve maternal and infant outcomes for pregnant women who are overweight or obese. Only two studies met selection criteria. A meta-analysis was not performed due to the considerable differences in study design between the two included studies. Nonetheless, no statistically significant differences were identified between the intervention and standard care groups for maternal or infant health outcomes. 

The Ronnberg review (2010) was undertaken to determine whether published trials of interventions to reduce excessive GWG are of sufficient quality and provide sufficient data to enable evidence-based recommendations to be developed for clinical practice in antenatal care. These authors concluded that as a consequence of important limitations in study design, inconsistency and lack of directness, the overall quality of evidence (as determined using the GRADE system) was judged to be very low and thus of insufficient quality to enable evidence-based recommendations to be developed for clinical practice in antenatal care [185]. Similarly the 2009 review by Bridsall, which assessed evidence for interventions to promote weight control or weight loss in women around the time of pregnancy, found there to be a deficiency of appropriately designed interventions for maternal obesity and highlighted areas for developing a more effective strategy [190].

The Skouteris review (2010) aimed to identify, and evaluate the effect of key variables designed to modify risk factors for excessive weight gain in pregnant women that have been targeted in interventions over the last decade [186]. While six of the included studies reported significantly less weight gain in the intervention women, only three showed that women in the intervention were significantly more likely to gain within recommended guidelines. The authors stipulate that findings were inconsistent in relation to what factors need to be targeted in intervention programs to reduce GWG and that consideration of psychological factors relevant to pregnancy, in addition to behavioural changes regarding eating and PA, should be considered. The 2010 meta-analyses of nine trials attempting to modulate diet and PA during pregnancy, performed by Streuling and colleagues, reported a significantly lower GWG in the intervention groups; a standardized mean difference of −0.22 units (95% CI: −0.38, −0.05 units) [187]. The authors concluded that interventions based on PA and dietary counseling, usually combined with supplementary weight monitoring, appear to be successful in reducing GWG. Additionally, this same group performed a second meta-analysis in 2011 which explored randomized controlled trials that intervened using PA only. These analyses found a mean difference in GWG of −0.61 (95% CI: −1.17, −0.06), suggesting less GWG in the intervention groups compared with the control groups [188]. The authors found no indication for publication bias in either review. In summary, these analyses suggest that physical activity during pregnancy might be successful in restricting GWG. 

The Tanentsapt (2011) review aimed to evaluate the effect of dietary interventions in reducing excessive GWG in normal, overweight and obese women, while also examining the impact of the interventions on maternal and child health outcomes [189]. There were 13 dietary intervention studies included in the review and 10 provided data for the analysis on total GWG. The interventions varied by design which included lifestyle counseling, calorie restriction, macronutrient composition, motivational phone calls, and feedback regarding weight gain. The review found that dietary interventions can reduce *total* GWG; a weighted mean difference of −1.92 (95% CI = −3.65, −0.19), but there was no significant evidence that dietary interventions can prevent *excessive* GWG. There was also evidence of reduced weight retention at six months postpartum with a weighted mean difference of −1.90 (95% CI = −2.69, −1.12), however, there were no significant effects on mean birth weight, pre-eclampsia, GDM and preterm birth [189]. Campbell *et al*.’s 2011 systematic review and meta-analysis of controlled trials of diet and PA interventions to prevent excessive weight gain during pregnancy also included a thematic synthesis of qualitative studies that investigated the views of women on weight management during pregnancy. The author’s overall conclusion was that despite intense and often tailored interventions, there was no statistically significant effect on weight gain during pregnancy (mean difference −0.28; 95% CI −0.64 to 0.09) [181]. Inadequate and often contradictory information regarding healthy weight management was reported by women in qualitative studies and this was addressed in the interventions but was insufficient to lead to reduced weight gain. Finally, the Quinlivan meta-analysis (2011) focused on dietary interventions aimed at restricting maternal weight gain in obese women and their affect on neonatal birth weight. This study examined four randomized controlled trials and identified that while there was a pooled mean difference in GWG of −6.5 kg (95% CI: −7.6 to −5.4 kg), there was no significant pool treatment effect for birth weight (p = 0.859) [184]. The authors concluded that it is possible to reduce GWG through antenatal dietary interventions without risking low neonatal birth weight, and that it may be effective for overweight or obese women to gain less weight than advised by the IOM.

Although weight-related outcomes tended to be more favourable and showed trends towards improvement for those in the intervention groups, indicating that interventions can help pregnant and postpartum women manage their weight, the conclusions put forth by the various reviews are inconsistent. The common thread that can be pulled from these reviews is that knowledge gaps remain regarding the benefits and potential harm associated with dietary and lifestyle interventions for overweight and obese pregnant women. Collectively, there is a consensus, recently echoed in the revised IOM Weight Gain During Pregnancy guidelines [42], that further evaluation through randomized trials with adequate power is required to demonstrate their efficacy with the hope that effective implementation in a clinical setting will help offset the many co-morbidities and poor health outcomes associated with maternal adiposity and downstream pediatric obesity [182,226,227].

Of specific interest to those of us engaged in the child obesity prevention effort is that none of the intervention studies outlined in Table 1 followed the offspring past delivery. Although some of the studies included in the aforementioned systematic reviews were able to limit excessive GWG or minimize post partum weight retention, there is a clear lack of trials addressing down-stream child growth and development outcomes resulting from maternal obesity and/or excessive GWG. Recently, the rationale to investigate intervention trials aimed at reducing excess weight gain during pregnancy has been reinforced by Stuebe *et al*. [228] who, for the first time in humans, linked maternal adiposity to offspring obesity in both child- and adult-hood.

There are several ongoing randomized trials examining the impact of interventions during the gestational period (LIMIT [229], NewLife(style) [230], FitFor2 [231], MOM [232], ETIP [233]) that may shed some light on potential barriers to, and effective strategies that promote optimal maternal-fetal outcomes. While GWG is a common target [229,230,233], other primary outcomes include insulin sensitivity in women at risk of GDM [231] and downstream offspring obesity [232]. However, all interventions ultimately aim to improve the health of mom and baby. 

## 4. Novel Mechanisms

**Figure 6:**
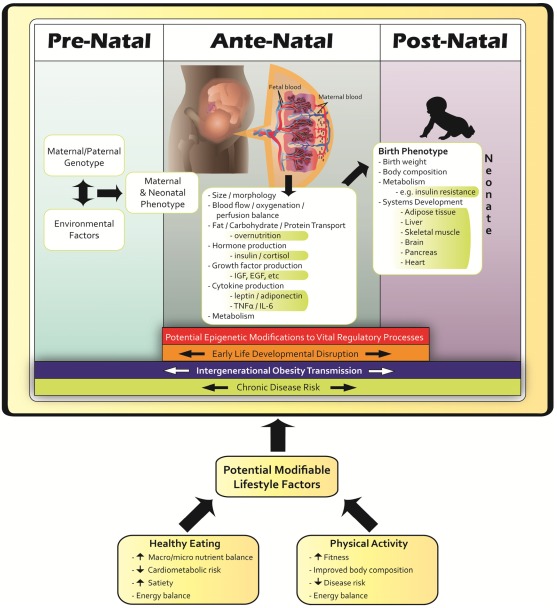
Complex relationships and potential role of various contributors to downstream obesity.

The mechanisms mediating the relationship between parental BMI and offspring BMI are not fully understood. However, an underlying genetic predisposition to positive energy balance as a result of familial risk factors has been identified [234]. It is reasonable to postulate that the rising incidence of obesity may be due to gene-environment interactions which predispose offspring to epigenetic modifications that alter the phenotype over time [94,235] (Figure 6). Of great interest is the idea that predisposition to over-consume energy (*i.e.*, hyperphagia) and engage in high levels of sedentary behaviour may be observed in offspring of women struggling with obesity, a finding documented in experimental animal models [236]. This observation aligns with Barker’s developmental origins of adult health and disease hypothesis [90,237,238,239], which originally linked poor nutrition *in utero* to chronic disease susceptibility and subsequent risk throughout the life course. Yet, in the context of over-nutrition, recent evidence supports the ideology that positive energy balance through maternal obesity and over-feeding can increase predisposition to offspring metabolic disease [157], an effect thought to be mediated by alterations in epigenetic regulation of metabolic pathways [111,240,241]. Given the increased prevalence of obesity and the intergenerational nature of this condition [66], attention has shifted to threats of over-nutrition during pregnancy as an important contributor to childhood adiposity and metabolic dysregulation later in life (Figure 6) [242,243,244,245]. The exact obesogenic factors leading to such disturbances are not well characterized but potential candidates include free fatty acids and/or triglycerides [87,88,89], or maternal hyperleptinemia, hyperglycemia, hyperinsulinemia and chronic low-grade maternal inflammation, as reviewed by Rooney and Ozanne [235]. Briefly, with respect to the inflammatory state characteristic of maternal obesity, it must be noted that human studies have shown strong associations between markers of oxidative stress (e.g., reactive oxygen species) and obesity, insulin resistance and T2D; with interference in insulin signaling acting as a mediating mechanism [246].

Of great interest is the observation that proinflammatory markers appear in liver and adipose tissue and precede the clinical presentation of insulin resistance [247] and may be suppressed with dietary restriction [248]. Furthermore, oxidative stressors during *in utero *development may affect the fetal-placental unit. For instance, placentas from obese gravidas show elevated expression of genes related to inflammation and oxidative stress [249] while fetal mesenchymal stem cell differentiation has been shown to be altered in a proinflammatory condition by preferentially shifting stem cell differentiation from a myogenic to adipogenic state [250]. Collectively, these findings suggest that an abnormal maternal milieu resulting from maternal obesity predispose aberrant embryonic and fetal development. Current evidence suggests that these events may be triggered by a proinflammatory state resulting in changes in gene expression and insulin resistance leading to the eventual presentation of an abnormal offspring phenotype [246]. Overall, despite animal [102,236,242] and human [234,242,251,252,253,254,255,256,257,258] studies that have examined the effects of maternal over-nutrition and obesity on offspring obesity and Type 2 diabetes, our knowledge concerning the precise mechanisms mediating childhood pathologies are far from complete. Knowing that the growing fetus receives its sustenance from maternal sources through the placenta, much attention has been directed to examining the role of this highly specialized organ on substrate transfer and subsequent fetal growth regulation in pregnancy. It is not unrealistic to presume that healthful eating and physical activity behaviours, that can change maternal metabolism and modify the availability of specific nutrients, could affect fetal body composition and downstream health. Even though the placenta has been implicated as a pivotal regulatory organ [259,260], few groups have explored placental mechanisms in pregnancies exposed to maternal exercise or complicated by diabetes [168,261,262,263,264,265,266,267,268,269]. Needless to say there is still much unchartered territory around substrate partitioning and nutrient transfer/transport in pregnancy in general, let alone those pregnancies complicated by maternal obesity and excessive GWG.

## 5. Conclusions

Children born to obese mothers or those who have experienced excessive GWG, have an increased risk of obesity themselves as a result of the likelihood of exposure to over-nutrition and associated developmental programming *in utero* as well as environmental exposure to the same obesogenic lifestyle as the mother. As such, targeting behaviours that lead to chronic exposure to energy surplus, and inappropriate levels of metabolic hormones *in utero *may potentially decrease susceptibility to downstream obesity and chronic disease and contribute to halting this intergenerational cycle. Given that pregnancy is the most a critical period of growth and development, and that minute changes in the intrauterine environment may have substantial impacts on health outcomes, it is of paramount importance that the underlying physiological factors involved with maternal-fetal obesity transmission are identified and that effective prevention and management strategies are designed. 

## References

[1] Thow A.M., Jan S., Leeder S., Swinburn B. (2010). The effect of fiscal policy on diet, obesity and chronic disease: A systematic review. Bull. World Health Organ..

[2] Shields M. (2006). Overweight and obesity among children and youth. Health Rep..

[3] Freedman D.S., Khan L.K., Dietz W.H., Srinivasan S.R., Berenson G.S. (2001). Relationship of childhood obesity to coronary heart disease risk factors in adulthood: The Bogalusa Heart Study. Pediatrics.

[4] Magarey A.M., Daniels L.A., Boulton T.J., Cockington R.A. (2003). Predicting obesity in early adulthood from childhood and parental obesity. Int. J. Obes. Relat. Metab. Disord..

[5] Must A. (2003). Does overweight in childhood have an impact on adult health?. Nutr. Rev..

[6] Freedman D.S., Dietz W.H., Srinivasan S.R., Berenson G.S. (1999). The relation of overweight to cardiovascular risk factors among children and adolescents: The Bogalusa Heart Study. Pediatrics.

[7] Fagot-Campagna A. (2000). Emergence of type 2 diabetes mellitus in children: Epidemiological evidence. J. Pediatr. Endocrinol. Metab..

[8] Sinha R., Fisch G., Teague B., Tamborlane W.V., Banyas B., Allen K., Savoye M., Rieger V., Taksali S., Barbetta G. (2002). Prevalence of impaired glucose tolerance among children and adolescents with marked obesity. N. Engl. J. Med..

[9] Eminoglu T.F., Camurdan O.M., Oktar S.O., Bideci A., Dalgic B. (2008). Factors related to non-alcoholic fatty liver disease in obese children. Turk. J. Gastroenterol..

[10] Tominaga K., Fujimoto E., Suzuki K., Hayashi M., Ichikawa M., Inaba Y. (2009). Prevalence of non-alcoholic fatty liver disease in children and relationship to metabolic syndrome, insulin resistance, and waist circumference. Environ. Health Prev. Med..

[11] Daniels S.R. (2006). The consequences of childhood overweight and obesity. Future Child..

[12] Kim S.Y., Dietz P.M., England L., Morrow B., Callaghan W.M. (2007). Trends in pre-pregnancy obesity in nine states, 1993–2003. Obesity (Silver Spring).

[13] Robinson H.E., O'Connell C.M., Joseph K.S., McLeod N.L. (2005). Maternal outcomes in pregnancies complicated by obesity. Obstet. Gynecol..

[14] Yogev Y., Catalano P.M. (2009). Pregnancy and obesity. Obstet. Gynecol. Clin. North Am..

[15] Hedley A.A., Ogden C.L., Johnson C.L., Carroll M.D., Curtin L.R., Flegal K.M. (2004). Prevalence of overweight and obesity among U.S. children, adolescents, and adults, 1999–2002. JAMA.

[16] Department of Health (2003). Health Survey for England 2002- Latest Trends.

[17] Ferraro Z.M., Barrowman N., Prud'homme D., Walker M., Wen S.W., Rodger M., Adamo K.B. (2011). Excessive gestational weight gain predicts large for gestational age neonates independent of maternal body mass index. J Matern.-Fetal Neonatal. Med..

[18] Gillman M.W., Rifas-Shiman S., Berkey C.S., Field A.E., Colditz G.A. (2003). Maternal gestational diabetes, birth weight, and adolescent obesity. Pediatrics.

[19] Parsons T.J., Power C., Manor O. (2001). Fetal and early life growth and body mass index from birth to early adulthood in 1958 British cohort: Longitudinal study. Br. Med. J..

[20] Whitaker R.C. (2004). Predicting preschooler obesity at birth: the role of maternal obesity in early pregnancy. Pediatrics.

[21] Oken E., Taveras E.M., Kleinman K.P., Rich-Edwards J.W., Gillman M.W. (2007). Gestational weight gain and child adiposity at age 3 years. Am. J. Obstet. Gynecol..

[22] Catalano P.M., Farrell K., Thomas A., Huston-Presley L., Mencin P., de Mouzon S.H., Amini S.B. (2009). Perinatal risk factors for childhood obesity and metabolic dysregulation. Am. J. Clin. Nutr..

[23] Whitaker R.C., Dietz W.H. (1998). Role of the prenatal environment in the development of obesity. J. Pediatr..

[24] Yu Z.B., Han S.P., Zhu G.Z., Zhu C., Wang X.J., Cao X.G., Guo X.R. (2011). Birth weight and subsequent risk of obesity: A systematic review and meta-analysis. Obes. Rev..

[25] Salihu H.M., Weldeselasse H.E., Rao K., Marty P.J., Whiteman V.E. (2011). The impact of obesity on maternal morbidity and feto-infant outcomes among macrosomic infants. J. Matern.-Fetal Neonatal. Med..

[26] Arendas K., Qiu Q., Gruslin A. (2008). Obesity in pregnancy: Pre-conceptional to postpartum consequences. J. Obstet. Gynaecol. Can..

[27] Guelinckx I., Devlieger R., Beckers K., Vansant G. (2008). Maternal obesity: Pregnancy complications, gestational weight gain and nutrition. Obes. Rev..

[28] Huda S.S., Brodie L.E., Sattar N. (2010). Obesity in pregnancy: Prevalence and metabolic consequences. Semin. Fetal. Neonatal. Med..

[29] Mehta S.H. (2008). Nutrition and pregnancy. Clin. Obstet. Gynecol..

[30] Satpathy H.K., Fleming A., Frey D., Barsoom M., Satpathy C., Khandalavala J. (2008). Maternal obesity and pregnancy. Postgrad. Med..

[31] Ong K.K., Ahmed M.L., Emmett P.M., Preece M.A., Dunger D.B. (2000). Association between postnatal catch-up growth and obesity in childhood: Prospective cohort study. Br. Med. J..

[32] Ong K.K. (2006). Size at birth, postnatal growth and risk of obesity. Horm. Res..

[33] Toschke A.M., Beyerlein A., von Kries R. (2005). Children at high risk for overweight: A classification and regression trees analysis approach. Obes. Res..

[34] Reilly J.J., Armstrong J., Dorosty A.R., Emmett P.M., Ness A., Rogers I., Steer C., Sherriff A. (2005). Early life risk factors for obesity in childhood: Cohort study. Br. Med. J..

[35] Karaolis-Danckert N., Buyken A.E., Kulig M., Kroke A., Forster J., Kamin W., Schuster A., Hornberg C., Keil T., Bergmann R.L., Wahn U., Lau S. (2008). How pre- and postnatal risk factors modify the effect of rapid weight gain in infancy and early childhood on subsequent fat mass development: Results from the Multicenter Allergy Study 90. Am. J. Clin. Nutr..

[36] Nader P.R., O'Brien M., Houts R., Bradley R., Belsky J., Crosnoe R., Friedman S., Mei Z., Susman E.J. (2006). Identifying risk for obesity in early childhood. Pediatrics.

[37] Guihard-Costa A.M., Papiernik E., Kolb S. (2004). Maternal predictors of subcutaneous fat in the term newborn. Acta Paediatr..

[38] Hull H.R., Thornton J.C., Ji Y., Paley C., Rosenn B., Mathews P., Navder K., Yu A., Dorsey K., Gallagher D. (2011). Higher infant body fat with excessive gestational weight gain in overweight women. Am. J. Obstet. Gynecol..

[39] Ludwig D.S., Currie J. (2010). The association between pregnancy weight gain and birthweight: A within-family comparison. Lancet.

[40] Schack-Nielsen L., Michaelsen K.F., Gamborg M., Mortensen E.L., Sorensen T.I. (2010). Gestational weight gain in relation to offspring body mass index and obesity from infancy through adulthood. Int. J. Obes. (Lond.).

[41] Wrotniak B.H., Shults J., Butts S., Stettler N. (2008). Gestational weight gain and risk of overweight in the offspring at age 7 y in a multicenter, multiethnic cohort study. Am. J. Clin. Nutr..

[42] Institute of Medicine (2009). Weight Gain During Pregnancy: Reexamining the Guidelines.

[43] Kinnunen T.I., Luoto R., Gissler M., Hemminki E. (2003). Pregnancy weight gain from 1960s to 2000 in Finland. Int. J. Obes. Relat. Metab. Disord..

[44] Weisman C.S., Hillemeier M.M., Downs D.S., Chuang C.H., Dyer A.M. (2010). Preconception predictors of weight gain during pregnancy: Prospective findings from the Central Pennsylvania Women's Health Study. Womens Health Issues.

[45] Olson C.M., Strawderman M.S. (2003). Modifiable behavioral factors in a biopsychosocial model predict inadequate and excessive gestational weight gain. J. Am. Diet. Assoc..

[46] Stotland N.E., Haas J.S., Brawarsky P., Jackson R.A., Fuentes-Afflick E., Escobar G.J. (2005). Body mass index, provider advice, and target gestational weight gain. Obstet. Gynecol..

[47] Wells C.S., Schwalberg R., Noonan G., Gabor V. (2006). Factors influencing inadequate and excessive weight gain in pregnancy: Colorado, 2000–2002. Matern. Child Health J..

[48] Gore S.A., Brown D.M., West D.S. (2003). The role of postpartum weight retention in obesity among women: A review of the evidence. Ann. Behav. Med..

[49] Cedergren M. (2006). Effects of gestational weight gain and body mass index on obstetric outcome in Sweden. Int. J. Gynaecol. Obstet..

[50] Cedergren M.I. (2007). Optimal gestational weight gain for body mass index categories. Obstet. Gynecol..

[51] Oken E., Kleinman K.P., Belfort M.B., Hammitt J.K., Gillman M.W. (2009). Associations of gestational weight gain with short- and longer-term maternal and child health outcomes. Am. J. Epidemiol..

[52] Park S., Sappenfield W.M., Bish C., Salihu H., Goodman D., Bensyl D.M. (2011). Assessment of the Institute of Medicine recommendations for weight gain during pregnancy: Florida, 2004–2007. Matern. Child Health J..

[53] Hinkle S.N., Sharma A.J., Dietz P.M. (2010). Gestational weight gain in obese mothers and associations with fetal growth. Am. J. Clin. Nutr..

[54] Catalano P.M., Ehrenberg H.M. (2006). The short- and long-term implications of maternal obesity on the mother and her offspring. BJOG.

[55] Ekblad U., Grenman S. (1992). Maternal weight, weight gain during pregnancy and pregnancy outcome. Int. J. Gynaecol. Obstet..

[56] Stevens-Simon C., McAnarney E.R. (1992). Determinants of weight gain in pregnant adolescents. J. Am. Diet. Assoc..

[57] Ananth C.V., Wen S.W. (2002). Trends in fetal growth among singleton gestations in the United States and Canada, 1985 through 1998. Semin. Perinatol..

[58] Choi S.K., Park I.Y., Shin J.C. (2011). The effects of pre-pregnancy body mass index and gestational weight gain on perinatal outcomes in Korean women: A retrospective cohort study. Reprod. Biol. Endocrinol..

[59] Saxena P., Tyagi S., Prakash A., Nigam A., Trivedi S.S. (2011). Pregnancy outcome of women with gestational diabetes in a tertiary level hospital of north India. Indian J. Community Med..

[60] Abrams B., Parker J.D. (1990). Maternal weight gain in women with good pregnancy outcome. Obstet. Gynecol..

[61] Gunderson E.P., Abrams B. (1999). Epidemiology of gestational weight gain and body weight changes after pregnancy. Epidemiol. Rev..

[62] Gunderson E.P., Abrams B., Selvin S. (2000). The relative importance of gestational gain and maternal characteristics associated with the risk of becoming overweight after pregnancy. Int. J. Obes. Relat. Metab. Disord..

[63] Keppel K.G., Taffel S.M. (1993). Pregnancy-related weight gain and retention: implications of the 1990 Institute of Medicine guidelines. Am. J. Public Health..

[64] McKeown T., Record R.G. (1957). The influence of weight and height on weight changes associated with pregnancy in women. J. Endocrinol..

[65] Luke B., Hediger M.L., Scholl T.O. (1996). Point of diminishing returns: When does gestational weight gain cease benefiting birthweight and begin adding to maternal obesity?. J. Matern. Fetal Med..

[66] Catalano P.M. (2003). Obesity and pregnancy—The propagation of a viscous cycle?. J. Clin. Endocrinol. Metab..

[67] Swinburn B.A., Sacks G., Hall K.D. (2011). The global obesity pandemic: Shaped by global drivers and local environments. Lancet.

[68] Shankar K., Harrell A., Liu X., Gilchrist J.M., Ronis M.J., Badger T.M. (2008). Maternal obesity at conception programs obesity in the offspring. Am. J. Physiol. Regul. Integr. Comp. Physiol..

[69] Villamor E., Cnattingius S. (2006). Interpregnancy weight change and risk of adverse pregnancy outcomes: A population-based study. Lancet.

[70] Ehrlich S.F., Hedderson M.M., Feng J., Davenport E.R., Gunderson E.P., Ferrara A. (2011). Change in body mass index between pregnancies and the risk of gestational diabetes in a second pregnancy. Obstet. Gynecol..

[71] Ehrenberg H.M., Huston-Presley L., Catalano P.M. (2003). The influence of obesity and gestational diabetes mellitus on accretion and the distribution of adipose tissue in pregnancy. Am. J. Obstet. Gynecol..

[72] Sohlstrom A., Wahlund L.O., Forsum E. (1993). Total body fat and its distribution during human reproduction as assessed by magnetic resonance imaging. Basic Life Sci..

[73] Kinoshita T., Itoh M. (2006). Longitudinal variance of fat mass deposition during pregnancy evaluated by ultrasonography: The ratio of visceral fat to subcutaneous fat in the abdomen. Gynecol. Obstet. Invest..

[74] Denison F.C., Roberts K.A., Barr S.M., Norman J.E. (2010). Obesity, pregnancy, inflammation, and vascular function. Reproduction.

[75] Giguere I., Prud'homme D., Strychar I., Doucet E., Adamo K.B. (2012). Gestational Weight gain, Post-partum Weight Loss or Retention, Body Composition and Metabolic profile of Pre-menopausal Women: A MONET Group Study. Am. J. Obstet. Gynecol..

[76] Lawlor D.A., Lichtenstein P., Fraser A., Langstrom N. (2011). Does maternal weight gain in pregnancy have long-term effects on offspring adiposity? A sibling study in a prospective cohort of 146,894 men from 136,050 families. Am. J. Clin. Nutr..

[77] O'Brien T.E., Ray J.G., Chan W.S. (2003). Maternal body mass index and the risk of preeclampsia: A systematic overview. Epidemiology.

[78] Frederick I.O., Rudra C.B., Miller R.S., Foster J.C., Williams M.A. (2006). Adult weight change, weight cycling, and prepregnancy obesity in relation to risk of preeclampsia. Epidemiology.

[79] Rudra C.B., Williams M.A., Lee I.M., Miller R.S., Sorensen T.K. (2005). Perceived exertion during prepregnancy physical activity and preeclampsia risk. Med. Sci. Sports Exerc..

[80] Sebire N.J., Jolly M., Harris J.P., Wadsworth J., Joffe M., Beard R.W., Regan L., Robinson S. (2001). Maternal obesity and pregnancy outcome: A study of 287,213 pregnancies in London. Int. J. Obes. Relat. Metab. Disord..

[81] Nucci L.B., Schmidt M.I., Duncan B.B., Fuchs S.C., Fleck E.T., Santos Britto M.M. (2001). Nutritional status of pregnant women: prevalence and associated pregnancy outcomes. Rev. Saude Publica.

[82] Callaway L.K., Prins J.B., Chang A.M., McIntyre H.D. (2006). The prevalence and impact of overweight and obesity in an Australian obstetric population. Med. J. Aust..

[83] Langer O., Yogev Y., Xenakis E.M., Brustman L. (2005). Overweight and obese in gestational diabetes: the impact on pregnancy outcome. Am. J. Obstet. Gynecol..

[84] Langer O., Yogev Y., Most O., Xenakis E.M. (2005). Gestational diabetes: the consequences of not treating. Am. J. Obstet. Gynecol..

[85] Catalano P.M., Hauguel-De M.S. (2011). Is it time to revisit the Pedersen hypothesis in the face of the obesity epidemic?. Am. J. Obstet. Gynecol..

[86] Silverman B.L., Rizzo T.A., Cho N.H., Metzger B.E. (1998). Long-term effects of the intrauterine environment. The Northwestern University Diabetes in Pregnancy Center. Diabetes Care.

[87] Kitajima M., Oka S., Yasuhi I., Fukuda M., Rii Y., Ishimaru T. (2001). Maternal serum triglyceride at 24–32 weeks' gestation and newborn weight in nondiabetic women with positive diabetic screens. Obstet. Gynecol..

[88] Knopp R.H., Bergelin R.O., Wahl P.W., Walden C.E. (1985). Relationships of infant birth size to maternal lipoproteins, apoproteins, fuels, hormones, clinical chemistries, and body weight at 36 weeks gestation. Diabetes.

[89] Nolan C.J., Riley S.F., Sheedy M.T., Walstab J.E., Beischer N.A. (1995). Maternal serum triglyceride, glucose tolerance, and neonatal birth weight ratio in pregnancy. Diabetes Care.

[90] Barker D.J. (1990). The fetal and infant origins of adult disease. Br. Med. J..

[91] Barker D.J., Bull A.R., Osmond C., Simmonds S.J. (1990). Fetal and placental size and risk of hypertension in adult life. BMJ.

[92] Barker D.J. (2000). *In utero* programming of cardiovascular disease. Theriogenology.

[93] Dabelea D., Hanson R.L., Lindsay R.S. (2000). Intrauterine exposure to diabetes conveys risks for type 2 diabetes and obesity: A study of discordant sibships. Diabetes.

[94] Gluckman P.D., Hanson M.A., Cooper C., Thornburg K.L. (2008). Effect of *in utero* and early-life conditions on adult health and disease. N. Engl. J. Med..

[95] Murtaugh M.A., Jacobs D.R., Moran A., Steinberger J., Sinaiko A.R. (2003). Relation of birth weight to fasting insulin, insulin resistance, and body size in adolescence. Diabetes Care.

[96] Wilkin T.J., Metcalf B.S., Murphy M.J., Kirkby J., Jeffery A.N., Voss L.D. (2002). The relative contributions of birth weight, weight change, and current weight to insulin resistance in contemporary 5-year-olds: The EarlyBird Study. Diabetes.

[97] Huang R.C., Burke V., Newnham J.P. (2007). Perinatal and childhood origins of cardiovascular disease. Int. J. Obes. (Lond.).

[98] Druet C., Ong K.K. (2008). Early childhood predictors of adult body composition. Best Pract. Res. Clin. Endocrinol. Metab..

[99] Ozanne S.E., Fernandez-Twinn D., Hales C.N. (2004). Fetal growth and adult diseases. Semin. Perinatol..

[100] Pettitt D.J., Jovanovic L. (2001). Birth weight as a predictor of type 2 diabetes mellitus: The U-shaped curve. Curr. Diab. Rep..

[101] Wei J.N., Sung F.C., Li C.Y. (2003). Low birth weight and high birth weight infants are both at an increased risk to have type 2 diabetes among schoolchildren in taiwan. Diabetes Care.

[102] Bayol S.A., Farrington S.J., Stickland N.C. (2007). A maternal 'junk food' diet in pregnancy and lactation promotes an exacerbated taste for 'junk food' and a greater propensity for obesity in rat offspring. Br. J. Nutr..

[103] Bayol S.A., Simbi B.H., Bertrand J.A., Stickland N.C. (2008). Offspring from mothers fed a 'junk food' diet in pregnancy and lactation exhibit exacerbated adiposity that is more pronounced in females. J. Physiol..

[104] Kral J.G., Biron S., Simard S. (2006). Large maternal weight loss from obesity surgery prevents transmission of obesity to children who were followed for 2 to 18 years. Pediatrics.

[105] Smith J., Cianflone K., Biron S. (2009). Effects of maternal surgical weight loss in mothers on intergenerational transmission of obesity. J. Clin. Endocrinol. Metab..

[106] Gluckman P.D., Pinal C.S. (2003). Regulation of fetal growth by the somatotrophic axis. J. Nutr..

[107] Gluckman P.D., Hanson M.A. (2008). Developmental and epigenetic pathways to obesity: an evolutionary-developmental perspective. Int. J. Obes. (Lond.).

[108] Gluckman P.D., Lillycrop K.A., Vickers M.H. (2007). Metabolic plasticity during mammalian development is directionally dependent on early nutritional status. Proc. Natl. Acad. Sci. USA.

[109] McMillen I.C., Rattanatray L., Duffield J.A. (2009). The early origins of later obesity: Pathways and mechanisms. Adv. Exp. Med. Biol..

[110] Levin B.E. (2008). Epigenetic influences on food intake and physical activity level: review of animal studies. Obesity (Silver Spring).

[111] Godfrey K.M., Sheppard A., Gluckman P.D. (2011). Epigenetic gene promoter methylation at birth is associated with child's later adiposity. Diabetes.

[112] Cawley J. (2010). The economics of childhood obesity. Health Aff. (Millwood).

[113] Olson C.M. (2005). Tracking of food choices across the transition to motherhood. J. Nutr. Educ. Behav..

[114] Phelan S. (2010). Pregnancy: A "teachable moment" for weight control and obesity prevention. Am. J. Obstet. Gynecol..

[115] Boney C.M., Verma A., Tucker R., Vohr B.R. (2005). Metabolic syndrome in childhood: Association with birth weight, maternal obesity, and gestational diabetes mellitus. Pediatrics.

[116] Finer L.B., Zolna M.R. (2011). Unintended pregnancy in the United States: Incidence and disparities, 2006. Contraception.

[117] Nohr E.A., Vaeth M., Baker J.L., Sorensen T.I., Olsen J., Rasmussen K.M. (2008). Combined associations of prepregnancy body mass index and gestational weight gain with the outcome of pregnancy. Am. J. Clin. Nutr..

[118] World Health Organization (2007). WHO European Ministerial Conference on Counteracting Obesity: Conference Report.

[119] Lau D.C., Douketis J.D., Morrison K.M., Hramiak I.M., Sharma A.M., Ur E. (2007). 2006 Canadian clinical practice guidelines on the management and prevention of obesity in adults and children [summary]. Can. Med. Assoc. J..

[120] Committee on the Impact of Pregnancy Weight on Maternal and Child Health (2007). Influence of Pregnancy Weight on Maternal and Child Health: Workshop Report.

[121] Butland B., Jebb S.A., Kopelman P. (2008). Foresight. Tackling Obesities: Future Choices.

[122] Krishnamoorthy U., Schram C.M., Hill S.R. (2006). Maternal obesity in pregnancy: Is it time for meaningful research to inform preventive and management strategies?. BJOG.

[123] Olafsdottir A.S., Skuladottir G.V., Thorsdottir I., Hauksson A., Steingrimsdottir L. (2006). Combined effects of maternal smoking status and dietary intake related to weight gain and birth size parameters. BJOG.

[124] King J.C. (2000). Physiology of pregnancy and nutrient metabolism. Am. J. Clin. Nutr..

[125] Anderson A.S. (2001). Symposium on 'nutritional adaptation to pregnancy and lactation'. Pregnancy as a time for dietary change?. Proc. Nutr. Soc..

[126] Weissgerber T.L., Wolfe L.A. (2006). Physiological adaptation in early human pregnancy: Adaptation to balance maternal-fetal demands. Appl. Physiol. Nutr. Metab..

[127] Butte N.F., Wong W.W., Treuth M.S., Ellis K.J., O'Brian S.E. (2004). Energy requirements during pregnancy based on total energy expenditure and energy deposition. Am. J. Clin. Nutr..

[128] Chen C.Y., Crott J., Liu Z., Smith D.E. (2010). Fructose and saturated fats predispose hyperinsulinemia in lean male rat offspring. Eur. J. Nutr..

[129] Ghezzi A.C., Cambri L.T., Ribeiro C., Botezelli J.D., Mello M.A. (2011). Impact of early fructose intake on metabolic profile and aerobic capacity of rats. Lipids Health Dis..

[130] Zhang Z.Y., Zeng J.J., Kjaergaard M. (2011). Effects of a maternal diet supplemented with chocolate and fructose beverage during gestation and lactation on rat dams and their offspring. Clin. Exp. Pharmacol. Physiol..

[131] Laraia B.A., Bodnar L.M., Siega-Riz A.M. (2007). Pregravid body mass index is negatively associated with diet quality during pregnancy. Public Health Nutr..

[132] Brock K., Huang W.Y., Fraser D.R. (2010). Low vitamin D status is associated with physical inactivity, obesity and low vitamin D intake in a large U.S. sample of healthy middle-aged men and women. J. Steroid Biochem. Mol. Biol..

[133] Mojtabai R. (2004). Body mass index and serum folate in childbearing age women. Eur. J. Epidemiol..

[134] Maghbooli Z., Hossein-Nezhad A., Karimi F., Shafaei A.R., Larijani B. (2008). Correlation between vitamin D3 deficiency and insulin resistance in pregnancy. Diabetes Metab. Res. Rev..

[135] Deierlein A.L., Siega-Riz A.M., Herring A. (2008). Dietary energy density but not glycemic load is associated with gestational weight gain. Am. J. Clin. Nutr..

[136] Crowther C.A., Hiller J.E., Moss J.R., McPhee A.J., Jeffries W.S., Robinson J.S. (2005). Effect of treatment of gestational diabetes mellitus on pregnancy outcomes. N. Engl. J. Med..

[137] Clapp J.F., Capeless E. (1991). The changing glycemic response to exercise during pregnancy. Am. J. Obstet. Gynecol..

[138] Clapp J.F. (1989). The effects of maternal exercise on early pregnancy outcome. Am. J. Obstet. Gynecol..

[139] Clapp J.F. (1991). Exercise and fetal health. J. Dev. Physiol..

[140] Davies G.A., Wolfe L.A., Mottola M.F., MacKinnon C. (2003). Joint SOGC/CSEP clinical practice guideline: Exercise in pregnancy and the postpartum period. Can. J. Appl. Physiol..

[141] Kramer M.S., McDonald S.W. (2006). Aerobic exercise for women during pregnancy. Cochrane Database Syst. Rev..

[142] Ferraro Z.M., Gaudet L., Adamo K.B. (2012). The potential impact of physical activity during pregnancy on maternal and neonatal outcomes. Obstet. Gynecol. Surv..

[143] Artal R. (2006). Outcome of fetuses in women with pregestational diabetes mellitus. J. Perinat. Med..

[144] Dyck R., Klomp H., Tan L.K., Turnell R.W., Boctor M.A. (2002). A comparison of rates, risk factors, and outcomes of gestational diabetes between aboriginal and non-aboriginal women in the Saskatoon health district. Diabetes Care.

[145] Artal R., Catanzaro R.B., Gavard J.A., Mostello D.J., Friganza J.C. (2007). A lifestyle intervention of weight-gain restriction: Diet and exercise in obese women with gestational diabetes mellitus. Appl. Physiol. Nutr. Metab..

[146] Dempsey J.C., Butler C.L., Sorensen T.K. (2004). A case-control study of maternal recreational physical activity and risk of gestational diabetes mellitus. Diabetes Res. Clin. Pract..

[147] Dempsey J.C., Sorensen T.K., Williams M.A. (2004). Prospective study of gestational diabetes mellitus risk in relation to maternal recreational physical activity before and during pregnancy. Am. J. Epidemiol..

[148] Ong M.J., Guelfi K.J., Hunter T., Wallman K.E., Fournier P.A., Newnham J.P. (2009). Supervised home-based exercise may attenuate the decline of glucose tolerance in obese pregnant women. Diabetes Metab..

[149] Saftlas A.F., Logsden-Sackett N., Wang W., Woolson R., Bracken M.B. (2004). Work, leisure-time physical activity, and risk of preeclampsia and gestational hypertension. Am. J. Epidemiol..

[150] Sorensen T.K., Williams M.A., Lee I.M., Dashow E.E., Thompson M.L., Luthy D.A. (2003). Recreational physical activity during pregnancy and risk of preeclampsia. Hypertension.

[151] Weissgerber T.L., Wolfe L.A., Davies G.A. (2004). The role of regular physical activity in preeclampsia prevention. Med. Sci. Sports Exerc..

[152] Dempsey J.C., Butler C.L., Williams M.A. (2005). No need for a pregnant pause: Physical activity may reduce the occurrence of gestational diabetes mellitus and preeclampsia. Exerc. Sport Sci. Rev..

[153] Osterdal M.L., Strom M., Klemmensen A.K. (2009). Does leisure time physical activity in early pregnancy protect against pre-eclampsia? Prospective cohort in Danish women. BJOG.

[154] Juhl M., Andersen P.K., Olsen J. (2008). Physical exercise during pregnancy and the risk of preterm birth: A study within the Danish National Birth Cohort. Am. J. Epidemiol..

[155] Penney D.S. (2008). The effect of vigorous exercise during pregnancy. J. Midwifery Womens Health.

[156] Artal R. (1992). Exercise and pregnancy. Clin. Sports Med..

[157] Hopkins S.A., Cutfield W.S. (2011). Exercise in pregnancy: Weighing up the long-term impact on the next generation. Exerc. Sport Sci. Rev..

[158] Florack E.I., Pellegrino A.E., Zielhuis G.A., Rolland R. (1995). Influence of occupational physical activity on pregnancy duration and birthweight. Scand. J. Work Environ. Health.

[159] Horns P.N., Ratcliffe L.P., Leggett J.C., Swanson M.S. (1996). Pregnancy outcomes among active and sedentary primiparous women. J. Obstet. Gynecol. Neonatal Nurs..

[160] Jarrett J.C., Spellacy W.N. (1983). Jogging during pregnancy: An improved outcome?. Obstet. Gynecol..

[161] Klebanoff M.A., Shiono P.H., Carey J.C. (1990). The effect of physical activity during pregnancy on preterm delivery and birth weight. Am. J. Obstet. Gynecol..

[162] Rabkin C.S., Anderson H.R., Bland J.M., Brooke O.G., Chamberlain G., Peacock J.L. (1990). Maternal activity and birth weight: a prospective, population-based study. Am. J. Epidemiol..

[163] Rose N., Haddow J., Palomaki G., Knight G. (1991). Self-rated physical activity level during the second trimester and pregnancy outcome. Obstet. Gynecol..

[164] Schramm W.F., Stockbauer J.W., Hoffman H.J. (1996). Exercise, employment, other daily activities, and adverse pregnancy outcomes. Am. J. Epidemiol..

[165] Snyder S., Pendergraph B. (2004). Exercise during pregnancy: What do we really know?. Am. Fam. Physician.

[166] Sternfeld B., Quesenberry C.P., Eskenazi B., Newman L.A. (1995). Exercise during pregnancy and pregnancy outcome. Med. Sci. Sports Exerc..

[167] Barakat R., Lucia A., Ruiz J.R. (2009). Resistance exercise training during pregnancy and newborn's birth size: A randomised controlled trial. Int. J. Obes. (Lond.).

[168] Clapp J.F. (2003). The effects of maternal exercise on fetal oxygenation and feto-placental growth. Eur. J. Obstet. Gynecol. Reprod. Biol..

[169] Jackson M.R., Gott P., Lye S.J., Ritchie J.W., Clapp J.F. (1995). The effects of maternal aerobic exercise on human placental development: Placental volumetric composition and surface areas. Placenta.

[170] Juhl M., Olsen J., Anderson P., Nohr E., Anderson A. (2010). Physical exercise during pregnancy and fetal growth measures: A study within the Danish National Birth Cohort. Am. J. Obstet. Gynecol..

[171] Clapp J.F., Capeless E. (1990). Neonatal morphometrics after endurance exercise during pregnancy. Am. J. Obstet. Gynecol..

[172] Bell R.J.P. (1995). The effect of vigorous exercise during pregnancy on birth-weight. Aust. N. Z. J. Obstet. Gynaecol..

[173] Campbell M., Mottola M.F. (2001). Recreational exercise and occupational activity during pregnancy and birth weight: A case-control study. Am. J. Obstet. Gynecol..

[174] Magann E., Evans S., Weitz B., Newnham J. (2002). Antepartum, intrapartum, and neonatal significance of exercise on healthy low-risk pregnant working women. Obstet. Gynecol..

[175] Rao S., Kanade A., Margetts B.M. (2003). Maternal activity in relation to birth size in rural India. The Pune Maternal Nutrition Study. Eur. J. Clin. Nutr..

[176] Clapp J.F., Kim H., Burciu B., Lopez B. (2000). Beginning regular exercise in early pregnancy: Effect on fetoplacental growth. Am. J. Obstet. Gynecol..

[177] Clapp J.F., Kim H., Burciu B., Schmidt S., Petry K., Lopez B. (2002). Continuing regular exercise during pregnancy: Effect of exercise volume on fetoplacental growth. Am. J. Obstet. Gynecol..

[178] Artal R., O'Toole M. (2003). Guidelines of the american college of obstetricians and gynecologists for exercise during pregnancy and the postpartum period. Br. J. Sports Med..

[179] Mottola M.F. (2009). Exercise prescription for overweight and obese women: Pregnancy and postpartum. Obstet. Gynecol. Clin. N. Am..

[180] Melzer K., Schutz Y., Boulvain M., Kayser B. (2010). Physical activity and pregnancy: Cardiovascular adaptations, recommendations and pregnancy outcomes. Sports Med..

[181] Campbell F., Johnson M., Messina J., Guillaume L., Goyder E. (2011). Behavioural interventions for weight management in pregnancy: A systematic review of quantitative and qualitative data. BMC Public Health.

[182] Dodd J.M., Crowther C.A., Robinson J.S. (2008). Dietary and lifestyle interventions to limit weight gain during pregnancy for obese or overweight women: A systematic review. Acta Obstet. Gynecol. Scand..

[183] Kuhlmann A.K., Dietz P.M., Galavotti C., England L.J. (2008). Weight-management interventions for pregnant or postpartum women. Am. J. Prev. Med..

[184] Quinlivan J.A., Julania S., Lam L. (2011). Antenatal dietary interventions in obese pregnant women to restrict gestational weight gain to institute of medicine recommendations: A meta-analysis. Obstet. Gynecol..

[185] Ronnberg A.K., Nilsson K. (2010). Interventions during pregnancy to reduce excessive gestational weight gain: A systematic review assessing current clinical evidence using the Grading of Recommendations, Assessment, Development and Evaluation (GRADE) system. BJOG.

[186] Skouteris H., Hartley-Clark L., McCabe M. (2010). Preventing excessive gestational weight gain: A systematic review of interventions. Obes. Rev..

[187] Streuling I., Beyerlein A., von Kries R. (2010). Can gestational weight gain be modified by increasing physical activity and diet counseling? A meta-analysis of interventional trials. Am. J. Clin. Nutr..

[188] Streuling I., Beyerlein A., Rosenfeld E., Hofmann H., Schulz T., von Kries R. (2011). Physical activity and gestational weight gain: A meta-analysis of intervention trials. BJOG.

[189] Tanentsapf I., Heitmann B.L., Adegboye A.R. (2011). Systematic review of clinical trials on dietary interventions to prevent excessive weight gain during pregnancy among normal weight, overweight and obese women. BMC Pregnancy Childbirth.

[190] Birdsall K.M., Vyas S., Khazaezadeh N., Oteng-Ntim E. (2009). Maternal obesity: A review of interventions. Int. J. Clin. Pract..

[191] Rae A., Bond D., Evans S.F., North F., Roberman B., Walters B. (2008). A randomized controlled trial of dietary energy restriction in the management of obese women with gestational diabetes. ANZJOG.

[192] Marquez-Sterling S., Perry A.C., Kaplan T.A., Halberstein R.A., Signorile J.F. (2000). Physical and psychological changes with vigorous exercise in sedentary primigravidae. Med. Sci. Sports Exerc..

[193] Polley B.A., Wing R.R., Sims C.J. (2002). Randomized controlled trial to prevent excessive weight gain in pregnant women. Int. J. Obes. Relat. Metab. Disord..

[194] Bechtel-Blackwell D.A. (2002). Computer-assisted self-interview and nutrition education in pregnant teens. Clin. Nurs. Res..

[195] Prevedel T., Calderon I., DeConti M., Consonni E., Rudge M. (2003). Maternal and perinatal effects of hydrotherapy in pregnancy. RGBO.

[196] Barakat R., Stirling J.R., Lucia A. (2008). Does exercise training during pregnancy affect gestational age? A randomised controlled trial. Br. J. Sports Med..

[197] Barakat R., Ruiz J.R., Stirling J.R., Zakynthinaki M., Lucia A. (2009). Type of delivery is not affected by light resistance and toning exercise training during pregnancy: A randomized controlled trial. Am. J. Obstet. Gynecol..

[198] Santos I.A., Stein R., Fuchs S.C. (2005). Aerobic exercise and submaximal functional capacity in overweight pregnant women: a randomized trial. Obstet. Gynecol..

[199] Garshasbi A., Faghih Z.S. (2005). The effect of exercise on the intensity of low back pain in pregnant women. Int. J. Gynaecol. Obstet..

[200] Hui A.L., Ludwig S., Gardiner P. (2006). Community-based exercise and dietary intervention during pregnancy: A pilot study. Can. J. Diabetes.

[201] Wolff S., Legarth J., Vangsgaard K., Toubro S., Astrup A. (2008). A randomized trial of the effects of dietary counseling on gestational weight gain and glucose metabolism in obese pregnant women. Int. J. Obes. (Lond ).

[202] Asbee S.M., Jenkins T.R., Butler J.R., White J., Elliot M., Rutledge A. (2009). Preventing excessive weight gain during pregnancy through dietary and lifestyle counseling: A randomized controlled trial. Obstet. Gynecol..

[203] Jeffries K., Shub A., Walker S.P., Hiscock R., Permezel M. (2009). Reducing excessive weight gain in pregnancy: A randomised controlled trial. Med. J. Aust..

[204] Thornton Y.S. (2009). Preventing excessive weight gain during pregnancy through dietary and lifestyle counseling: A randomized controlled trial. Obstet. Gynecol..

[205] Landon M.B., Spong C.Y., Thom E. (2009). A multicenter, randomized trial of treatment for mild gestational diabetes. N. Engl. J. Med..

[206] Baciuk E.P., Pereira R.I., Cecatti J.G., Braga A.F., Cavalcante S.R. (2008). Water aerobics in pregnancy: Cardiovascular response, labor and neonatal outcomes. Reprod. Health.

[207] Cavalcante S.R., Cecatti J.G., Pereira R.I., Baciuk E.P., Bernardo A.L., Silveira C. (2009). Water aerobics II: maternal body composition and perinatal outcomes after a program for low risk pregnant women. Reprod. Health.

[208] Guelinckx I., Devlieger R., Mullie P., Vansant G. (2010). Effect of lifestyle intervention on dietary habits, physical activity, and gestational weight gain in obese pregnant women: A randomized controlled trial. Am. J. Clin. Nutr..

[209] Hopkins S.A., Baldi J.C., Cutfield W.S., McCowan L., Hofman P.L. (2010). Exercise training in pregnancy reduces offspring size without changes in maternal insulin sensitivity. J. Clin. Endocrinol. Metab..

[210] Korpi-Hyovalti E.A., Laaksonen D.E., Schwab U.S. (2011). Feasibility of a lifestyle intervention in early pregnancy to prevent deterioration of glucose tolerance. BMC Public Health.

[211] Hui A., Back L., Ludwig S. (2012). Lifestyle intervention on diet and exercise reduced excessive gestational weight gain in pregnant women under a randomised controlled trial. BJOG.

[212] Luoto R., Kinnunen T.I., Aittasalo M. (2011). Primary prevention of gestational diabetes mellitus and large-for-gestational-age newborns by lifestyle counseling: A cluster-randomized controlled trial. PLoS Med..

[213] Phelan S., Phipps M.G., Abrams B., Darroch F., Schaffner A., Wing R.R. (2011). Randomized trial of a behavioral intervention to prevent excessive gestational weight gain: The Fit for Delivery Study. Am. J. Clin. Nutr..

[214] Quinlivan J.A., Lam L.T., Fisher J. (2011). A randomised trial of a four-step multidisciplinary approach to the antenatal care of obese pregnant women. Aust. N. Z. J. Obstet. Gynaecol..

[215] Nascimento S.L., Surita F.G., Parpinelli M.A., Siani S., Pinto e Silva J.L. (2011). The effect of an antenatal physical exercise programme on maternal/perinatal outcomes and quality of life in overweight and obese pregnant women: A randomised clinical trial. BJOG.

[216] Haakstad L.A., Bo K. (2011). Effect of regular exercise on prevention of excessive weight gain in pregnancy: A randomised controlled trial. Eur. J. Contracept. Reprod. Health Care.

[217] Haakstad L.A., Bo K. (2011). Exercise in pregnant women and birth weight: A randomized controlled trial. BMC Pregnancy Childbirth.

[218] Vinter C.A., Jensen D.M., Ovesen P., Beck-Nielsen H., Jorgensen J.S. (2011). The LiP (Lifestyle in Pregnancy) study: A randomized controlled trial of lifestyle intervention in 360 obese pregnant women. Diabetes Care.

[219] Gray-Donald K., Robinson E., Collier A., David K., Renaud L., Rodrigues S. (2000). Intervening to reduce weight gain in pregnancy and gestational diabetes mellitus in Cree communities: An evaluation. CMAJ.

[220] Olson C.M., Strawderman M.S., Reed R.G. (2004). Efficacy of an intervention to prevent excessive gestational weight gain. Am. J. Obstet. Gynecol..

[221] Kinnunen T.I., Pasanen M., Aittasalo M. (2007). Preventing excessive weight gain during pregnancy - a controlled trial in primary health care. Eur. J. Clin. Nutr..

[222] Claesson I.M., Sydsjo G., Brynhildsen J. (2008). Weight gain restriction for obese pregnant women: A case-control intervention study. BJOG.

[223] Shirazian T., Monteith S., Friedman F., Rebarber A. (2010). Lifestyle modification program decreases pregnancy weight gain in obese women. Am. J. Perinatol..

[224] Mottola M.F., Giroux I., Gratton R. (2010). Nutrition and exercise prevent excess weight gain in overweight pregnant women. Med. Sci. Sports Exerc..

[225] Lindholm E.S., Norman M., Kilander C.P., Altman D. (2010). Weight control program for obese pregnant women. Acta Obstet. Gynecol. Scand..

[226] Olson C.M. (2007). A call for intervention in pregnancy to prevent maternal and child obesity. Am. J. Prev. Med..

[227] Gavard J.A., Artal R. (2008). Effect of exercise on pregnancy outcome. Clin. Obstet. Gynecol..

[228] Stuebe A.M., Forman M.R., Michels K.B. (2009). Maternal-recalled gestational weight gain, pre-pregnancy body mass index, and obesity in the daughter. Int. J. Obes. (Lond.).

[229] Dodd J.M., Turnbull D.A., McPhee A.J., Wittert G., Crowther C.A., Robinson J.S. (2011). Limiting weight gain in overweight and obese women during pregnancy to improve health outcomes: The LIMIT randomised controlled trial. BMC Pregnancy Childbirth.

[230] Althuizen E., van Poppel M.N., Seidell J.C., van der Wijden C., van Mechelen W. (2006). Design of the New Life(style) study: A randomised controlled trial to optimise maternal weight development during pregnancy. [ISRCTN85313483]. BMC Public Health..

[231] Oostdam N., van Poppel M.N., Eekhoff E.M., Wouters M.G., van Mechelen W. (2009). Design of FitFor2 study: The effects of an exercise program on insulin sensitivity and plasma glucose levels in pregnant women at high risk for gestational diabetes. BMC Pregnancy Childbirth.

[232] Adamo K.B., Ferraro Z., Rutherford J. (2010). The maternal obesity management (MOM) trial: A lifestyle intervention during pregnancy to minimize downstream obesity. Appl. Physiol. Nutr. Metab..

[233] Moholdt T.T., Salvesen K., Ingul C.B., Vik T., Oken E., Morkved S. (2011). Exercise Training in Pregnancy for obese women (ETIP): Study protocol for a randomised controlled trial. Trials.

[234] Lee J.H., Reed D.R., Price R.A. (1997). Familial risk ratios for extreme obesity: Implications for mapping human obesity genes. Int. J. Obes. Relat. Metab. Disord..

[235] Rooney K., Ozanne S.E. (2011). Maternal over-nutrition and offspring obesity predisposition: Targets for preventative interventions. Int. J. Obes. (Lond.).

[236] Samuelsson A.M., Matthews P.A., Argenton M. (2008). Diet-induced obesity in female mice leads to offspring hyperphagia, adiposity, hypertension, and insulin resistance: A novel murine model of developmental programming. Hypertension..

[237] Barker D.J., Osmond C., Golding J., Kuh D., Wadsworth M.E. (1989). Growth in utero, blood pressure in childhood and adult life, and mortality from cardiovascular disease. Br. Med. J..

[238] Barker D.J., Gluckman P.D., Godfrey K.M., Harding J.E., Owens J.A., Robinson J.S. (1993). Fetal nutrition and cardiovascular disease in adult life. Lancet.

[239] Barker D.J. (1995). Fetal origins of coronary heart disease. Br. Med. J..

[240] Godfrey K.M., Inskip H.M., Hanson M.A. (2011). The long-term effects of prenatal development on growth and metabolism. Semin. Reprod. Med..

[241] Hanson M., Godfrey K.M., Lillycrop K.A., Burdge G.C., Gluckman P.D. (2011). Developmental plasticity and developmental origins of non-communicable disease: Theoretical considerations and epigenetic mechanisms. Prog. Biophys. Mol. Biol..

[242] Ainge H., Thompson C., Ozanne S.E., Rooney K.B. (2011). A systematic review on animal models of maternal high fat feeding and offspring glycaemic control. Int. J. Obes. (Lond.).

[243] George L.A., Uthlaut A.B., Long N.M. (2010). Different levels of overnutrition and weight gain during pregnancy have differential effects on fetal growth and organ development. Reprod. Biol. Endocrinol..

[244] Muhlhausler B.S., Adam C.L., Findlay P.A., Duffield J.A., McMillen I.C. (2006). Increased maternal nutrition alters development of the appetite-regulating network in the brain. FASEB J..

[245] Wang J., Ma H., Tong C. (2010). Overnutrition and maternal obesity in sheep pregnancy alter the JNK-IRS-1 signaling cascades and cardiac function in the fetal heart. FASEB J..

[246] Alfaradhi M.Z., Ozanne S.E. (2011). Developmental programming in response to maternal overnutrition. Front. Genet..

[247] Matsuzawa-Nagata N., Takamura T., Ando H. (2008). Increased oxidative stress precedes the onset of high-fat diet-induced insulin resistance and obesity. Metabolism.

[248] Dandona P., Mohanty P., Ghanim H. (2001). The suppressive effect of dietary restriction and weight loss in the obese on the generation of reactive oxygen species by leukocytes, lipid peroxidation, and protein carbonylation. J. Clin. Endocrinol. Metab..

[249] Radaelli T., Varastehpour A., Catalano P., Hauguel-De M.S. (2003). Gestational diabetes induces placental genes for chronic stress and inflammatory pathways. Diabetes.

[250] Tong J.F., Yan X., Zhu M.J., Ford S.P., Nathanielsz P.W., Du M. (2009). Maternal obesity downregulates myogenesis and beta-catenin signaling in fetal skeletal muscle. Am. J. Physiol. Endocrinol. Metab..

[251] Barker D.J. (2004). The developmental origins of chronic adult disease. Acta Paediatr. Suppl..

[252] Fraser A., Tilling K., Donald-Wallis C. (2010). Association of maternal weight gain in pregnancy with offspring obesity and metabolic and vascular traits in childhood. Circulation.

[253] Gale C.R., Javaid M.K., Robinson S.M., Law C.M., Godfrey K.M., Cooper C. (2007). Maternal size in pregnancy and body composition in children. J. Clin. Endocrinol. Metab..

[254] Lawlor D.A., Smith G.D., O'Callaghan M. (2007). Epidemiologic evidence for the fetal overnutrition hypothesis: Findings from the mater-university study of pregnancy and its outcomes. Am. J. Epidemiol..

[255] Lawlor D.A., Fraser A., Lindsay R.S. (2010). Association of existing diabetes, gestational diabetes and glycosuria in pregnancy with macrosomia and offspring body mass index, waist and fat mass in later childhood: Findings from a prospective pregnancy cohort. Diabetologia.

[256] McMillen I.C., MacLaughlin S.M., Muhlhausler B.S., Gentili S., Duffield J.L., Morrison J.L. (2008). Developmental origins of adult health and disease: The role of periconceptional and foetal nutrition. Basic Clin. Pharmacol. Toxicol..

[257] Metzger B.E., Lowe L.P., Dyer A.R. (2008). Hyperglycemia and adverse pregnancy outcomes. N. Engl. J. Med..

[258] Whitaker R.C., Wright J.A., Pepe M.S., Seidel K.D., Dietz W.H. (1997). Predicting obesity in young adulthood from childhood and parental obesity. N. Engl. J. Med..

[259] Bauer M.K., Harding J.E., Bassett N.S. (1998). Fetal growth and placental function. Mol. Cell. Endocrinol..

[260] Rahnama F., Shafiei F., Gluckman P.D., Mitchell M.D., Lobie P.E. (2006). Epigenetic regulation of human trophoblastic cell migration and invasion. Endocrinology.

[261] Clapp J.F. (2006). Influence of endurance exercise and diet on human placental development and fetal growth. Placenta.

[262] Clapp J.F., Schmidt S., Paranjape A., Lopez B. (2004). Maternal insulin-like growth factor-I levels (IGF-I) reflect placental mass and neonatal fat mass. Am. J. Obstet. Gynecol..

[263] Jansson T., Wennergren M., Illsley N.P. (1993). Glucose transporter protein expression in human placenta throughout gestation and in intrauterine growth retardation. J. Clin. Endocrinol. Metab..

[264] Jansson T., Wennergren M., Powell T.L. (1999). Placental glucose transport and GLUT 1 expression in insulin-dependent diabetes. Am. J. Obstet. Gynecol..

[265] Jansson T., Ekstrand Y., Wennergren M., Powell T.L. (2001). Placental glucose transport in gestational diabetes mellitus. Am. J. Obstet. Gynecol..

[266] Jansson T., Ylven K., Wennergren M., Powell T.L. (2002). Glucose transport and system A activity in syncytiotrophoblast microvillous and basal plasma membranes in intrauterine growth restriction. Placenta.

[267] Jansson T., Ekstrand Y., Bjorn C., Wennergren M., Powell T.L. (2002). Alterations in the activity of placental amino acid transporters in pregnancies complicated by diabetes. Diabetes.

[268] Jansson T., Powell T.L. (2006). IFPA 2005 Award in Placentology Lecture. Human placental transport in altered fetal growth: does the placenta function as a nutrient sensor?—A review. Placenta.

[269] Roos S., Powell T.L., Jansson T. (2009). Placental mTOR links maternal nutrient availability to fetal growth. Biochem. Soc. Trans..

